# Exploratory Study of Web-Based Planning and Mobile Text Reminders in an Overweight Population

**DOI:** 10.2196/jmir.1773

**Published:** 2011-12-20

**Authors:** Anastasia Soureti, Peter Murray, Mark Cobain, Mai Chinapaw, Willem van Mechelen, Robert Hurling

**Affiliations:** ^1^Unilever DiscoverBedforshireUnited Kingdom; ^2^Department of Public and Occupational Health and EMGO Institute for Health and Care ResearchVU University Medical CentreAmsterdamNetherlands

**Keywords:** Implementation intentions, mobile text reminders, saturated fat

## Abstract

**Background:**

Forming specific health plans can help translate good intentions into action. Mobile text reminders can further enhance the effects of planning on behavior.

**Objective:**

Our aim was to explore the combined impact of a Web-based, fully automated planning tool and mobile text reminders on intention to change saturated fat intake, self-reported saturated fat intake, and portion size changes over 4 weeks.

**Methods:**

Of 1013 men and women recruited online, 858 were randomly allocated to 1 of 3 conditions: a planning tool (PT), combined planning tool and text reminders (PTT), and a control group. All outcome measures were assessed by online self-reports. Analysis of covariance was used to analyze the data.

**Results:**

Participants allocated to the PT (mean_sat_ _urated_ _fat_ 3.6, mean_copingplanning_ 3) and PTT (mean_saturatedfat_ 3.5, mean_copingplanning_ 3.1) reported a lower consumption of high-fat foods (*F*_2,571_ = 4.74, *P* = .009) and higher levels of coping planning (*F*_2,571_ = 7.22, *P* < .001) than the control group (mean_sat_ _urated__f__at_ 3.9, mean_copingplanning_ 2.8). Participants in the PTT condition also reported smaller portion sizes of high-fat foods (mean 2.8; *F*_2,__569_ = 4.12, *P* = .0) than the control group (mean_portions_ 3.1). The reduction in portion size was driven primarily by the male participants in the PTT (*P* = .003). We found no significant group differences in terms of percentage saturated fat intake, intentions, action planning, self-efficacy, or feedback on the intervention.

**Conclusions:**

These findings support the use of Web-based tools and mobile technologies to change dietary behavior. The combination of a fully automated Web-based planning tool with mobile text reminders led to lower self-reported consumption of high-fat foods and greater reductions in portion sizes than in a control condition.

**Trial Registration:**

International Standard Randomized Controlled Trial Number (ISRCTN): 61819220; http://www.controlled-trials.com/ISRCTN61819220 (Archived by WebCite at http://www.webcitation.org/63YiSy6R8)

## Introduction

A healthy diet low in saturated fat is a popular recommendation in helping overweight and obese individuals eat healthier and reduce their health risk factors [1]. Since sustained change is very difficult, it is important to identify approaches that help people maintain new healthy behaviors once initiated. We have used the Health Action Process Approach model [2-4] to explore behavioral change, as it provides a theoretical framework on how to maintain the translation of intentions into action.

Since good intentions are not always translated into action, the emphasis was on using specific plans also known as implementation intentions. A meta-analysis of 94 studies [5] showed that implementation intentions had a positive effect of medium to large magnitude on goal achievement. Several different formats have been used in the past [6-8]. We used the *i* *f* *...* *then* format in the present study. The *i* *f* statements are purported to increase the accessibility of critical situations to a person, while the *then* component of the plan creates a stronger link between the situational cue and the goal-directed response. Promising results of this format have been reported in several health-related behaviors [9-12].

Most of the studies on implementation intentions have used face-to-face communication. These studies showed a reliable effect on diet, physical activity, and smoking cessation [5,8-11], whereas evidence from the two studies on the effects of Web-based interventions was mixed [12,13]. In a study conducted in an occupational setting, use of Web-based implementation intentions backfired, such that participants who did not form an implementation intention exercised significantly more than participants who formed an implementation intention [13]. In a recent dietary intervention, participants allocated to a Web-based implementation intention condition reported a reduction in their self-reported saturated fat intake [12].

Reminding people of their plans could enhance the impact of implementation intentions on behavior [14,15]. Delivering strategies via mobile phone technology is particularly appealing because of the widespread use of mobiles phones in the United Kingdom (where this study is being conducted), Europe, and the United States [16,17]. Penetration in Europe has surpassed the 100% mark [18]. Although the application of mobile and text-based technology for behavior change is in its infancy, there is some supportive evidence for physical activity, exercise behavior, and smoking cessation [19-24]. In one of these studies aimed at improving exercise behavior [19], participants were randomly allocated to 1 of 5 conditions (implementation intentions and text reminders, implementation intentions, text reminders, and 1 of 2 control groups). In the follow-up 4 weeks later, results suggested a superiority of the combined condition in the frequency of exercise, while neither the text reminders nor the implementation intention conditions alone were effective. In a later study, the same authors discussed that pairing implementation intentions with goal reminders increased the level of brisk walking and appeared to activate other related health behaviors such as weight loss [20].

### Objectives

The primary aim of the present study was to assess the effects of a fully automated planning tool and mobile text reminders on participants’ reduced intake of high-fat foods and food portion sizes. We hypothesized that participants in the combined planning tool and text reminders (PTT) condition would reduce their consumption of high-fat foods and make greater changes in their portion sizes. A secondary aim of this study was to assess changes in other health-related behaviors not directly targeted by the intervention. We also investigated the intervention effects on social cognitive measures (eg, self-efficacy, planning, and intentions) and the mediating properties of planning.

## Methods

### Participants

Participants were recruited by a recruitment agency via an online questionnaire that screened them for eligibility. The self-reported eligibility criteria were age (30–60 years), weight (body mass index [BMI] >25 kg/m^2^), not having a diagnosis of a heart condition or cancer, not being pregnant, and being a mobile phone user, happy to receive text reminders, computer savvy, and motivated to change their dietary patterns. Only motivated individuals were included, since they are closer to enacting their behavior and are known to make better use of a planning regimen [25,26]. We also chose overweight participants because, though at no immediate risk of disease, they would be more likely than normal-weight individuals to benefit from planning and saturated fat reductions. We selected individuals in the age range between 30 and 60 years because this is the age when they are starting to get more interested in their long-term health. Allocation of the participants to the 3 conditions at the intervention stage was stratified by age group (30–45 years or 46–60 years) and gender.

### Design and Procedures

This study was conducted between January and March 2010 and was registered retrospectively (ISRCTN61819220). This was an exploratory randomized controlled, between-groups study with no participant–experimenter contact. Participants were given online instructions and completed each week’s session from the convenience of their home computer. At week 1, participants were recruited by an online agency, signed an online consent form [27] (Multimedia Appendix 1), and were then randomly allocated into 1 of 3 conditions using a computer-generated list of random numbers: (1) control group, (2) planning tool (PT), and (3) PTT. Participants in the PT condition logged in to a Web-based, fully automated program, where they were prompted to identify a list of situations to change their saturated fat intake. The program then guided them to match these situations with a list of behaviors. They completed the session once and were not able to revisit the website to make any changes, such as to create more plans. Participants in the PTT condition first used the planning tool and then were offered text reminders of their plans. All 3 groups received educational information on the importance of a healthy diet low in saturated fat, and the association between high cholesterol and being overweight was highlighted (Multimedia Appendix 1). The term bad fats was used to refer to saturated fat. At the end of week 1, all participants completed an online questionnaire on their current saturated fat intake, maintenance self-efficacy, and intentions to change their dietary intake. A coupon for a cholesterol-lowering product, with £0.50 off the face value, was mailed to all participants as a reward for successfully completing baseline assessments.

Participants revisited the website 4 weeks later (week 5) and completed a follow-up online questionnaire on maintenance and recovery self-efficacy, action and coping planning, saturated fat intake, and portion size changes. Participants received £15 upon study completion and were entered in a prize draw for vouchers (£200 value).

### Interventions

#### The Planning Tool

Participants who received the planning tool selected from a list of 13 situations, in which they were tempted to eat unhealthily and then chose an approach to change their behavior from a list of 13 solutions. The solutions were based on constructs from the Processes of Change Model (eg, counterconditioning, stimulus control, and helpful relationships) [28]. Several nutritionally based behaviors were also included from an accredited site [29] after review by an expert nutritionist. The list of situations consisted of both situational cues (eg, having lunch) focusing on the *when and where* and motivational cues (eg, feeling bored) linked to the reasons (*why*) for performing a specific behavior [30]. Motivational cues were divided into 3 main situations: (1) experiencing positive affect, (2) experiencing negative affect, and (3) being faced with cravings [31,32]. The situations were translated into *if* statements (eg, “If I’m having breakfast”) and the solutions were translated into *then* statements (eg, “then I will tell myself I can eat healthily”).

**Figure 1:**
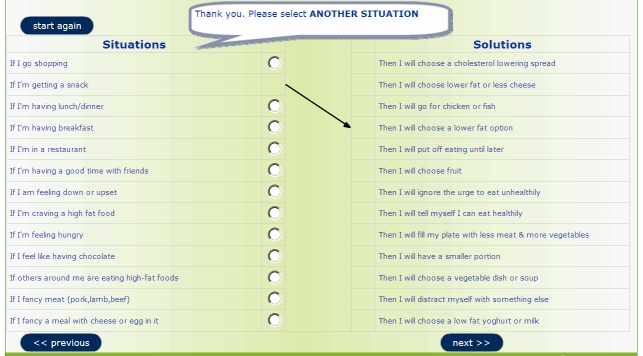
Web-based, fully automated planning tool for changing dietary behavior.

For every situation–solution pair chosen, the program drew a line on the computer screen to visually link the two together [10]. Participants were asked to complete at least 3 situation-solution pairs. Once these pairs were chosen and saved, participants were not able to revisit the program to change them during the 4-week period. The planning tool is shown in Figure 1.

#### Text Reminders

After completing the planning session, participants in the PTT entered their mobile number and chose a time band to receive text reminders of their plans.

#### Control Group

At the end of the study, participants in the control group received educational information on the importance of a healthy diet low in saturated and on the association between high cholesterol and being overweight. At weeks 1 and 5, they filled out the same online questionnaires as the participants in the rest of the experimental conditions .

### Outcome Measures and Statistical Considerations

#### Behavioral Outcomes

Behavior was assessed by the following measures.

(1) A *food frequency questionnaire* [33], which records the frequency of consumption of 63 common foods. This food frequency questionnaire has good test–retest reliability (*r* = .62, *P* < .01) [33] and validity when compared with 10-day weighed records [33-35].

(2) A *2* *-* *item scale* (*r* = .79, *P* < .001) adapted from a previous study [34]. Participants were asked to report on a 7-point Likert scale their agreement on consumption of high-saturated fat foods (“I have eaten foods high in bad fats in the last week”) followed by frequency of consumption of these foods (“How often did you eat foods high in bad fats in the last week?”).

(3) *Portion size changes* in the consumption of 11 items (eg, meat dishes, whole milk, bacon, ordinary cheese, chocolate, chips). These items accounted for the highest reported saturated fat intake in the food frequency questionnaire from a previous study [12]. Participants were asked to report changes in their portion sizes on a 7-point Likert scale (from a lot less to a lot more). The items were highly correlated with each other (*r* = .92) and were analyzed together as a composite score.

(4) *Other health behav* *ior* *s* measured by 6 items (Cronbach alpha = .778) on a 7-point Likert scale (from a lot less to a lot more). Participants were asked to report changes in other health areas, namely alcohol, use of cholesterol-lowering products, weight changes, smoking, physical activity, and eating a well-balanced diet.

#### Social Cognitive Outcomes

(1) *Intentions* to reduce consumption of high-fat foods were assessed on a 7-point Likert scale by 3 items (eg, “I intend to eat smaller portions of high-fat foods, replace high fat with low-fat alternatives”). Due to good reliability (*r* = .88), the mean of the 3 items was used in the analysis.

(2) *Maintenance and r* *ecovery s* *elf-efficacy* were modified from previous research [3 4,36,37]. Maintenance self-efficacy (Cronbach alpha = .79), which was measured at all study times, consisted of 3 items focusing on confidence at sustaining change in the face of difficulties. It consisted of items such as “I am certain that I could overcome difficulties when trying to eat more healthily even if...I don’t see success at once, I won’t get support for my first attempts.” Recovery self-efficacy (Spearman *r* = .67) was assessed only at week 5. It consisted of 2 items exploring confidence to start eating healthily when lapses occur. Items were measured on a 4-point scale (not at all, barely true, mostly true, exactly true).

(3) *Action and c* *oping p* *lanning* were adapted from previous research [3,4,36-39]. Action planning consisted of 2 items (“I now have my own plan regarding a) when and b) how to eat more healthily”) (Spearman *r* = .77). Coping planning consisted of 3 items (Cronbach alpha = .84) and focused on having a plan to deal with barriers (eg, “I have a detailed plan how to avoid high-risk situations where the urge to eat unhealthy food is high”).

(4) *Feedback on the i* *ntervention* was assessed at week 5 on a 7-point Likert scale (strongly disagree to strongly agree). Participants were asked to rate the intervention in terms of its personal relevance, interest, trustworthiness, credibility, feelings of enjoyment, and worry. All items were adapted from previous studies [40-42].

#### Statistical Considerations

This was an intention-to-treat analysis based on those participants who successfully completed week 1 assessments (n = 808). Data from the food frequency questionnaire were summarized to yield the total calorie intake per participant and the percentage of total energy intake due to saturated fat. Analysis of variance with baseline covariates was conducted to analyze the behavioral (2-item scale, food frequency questionnaire, portion sizes changes, and other health behaviors) and social cognitive measures (intentions, self-efficacy, planning, and feedback). Baseline self-reported saturated fat intake was included as a covariate for the analysis of the primary outcome measures. Other potential covariates (eg, smoking, BMI, and social economic status) were retained if significant in the analysis. For any significant condition effects, we conducted Tukey–Kramer-adjusted multiple comparisons to test for further differences between the groups. All of the above analysis was carried out using version 9.2 of SAS software (SAS Institute, Cary, NC, USA). To test the mediating properties of planning, we used the SPSS macro developed by Preacher and Hayes [43]. This program uses a bootstrapping resampling strategy to evaluate significance of the model and effects of the mediator; for this analysis, 5000 bootstrap samples were used [33-35]. In the following steps, we assessed (1) the relationship between the intervention and the 2-item scale, (2) the relationship between the intervention and planning, and 3) the relationship between the intervention and the 2-item scale, taking into account planning. The bootstrapped a*b path of planning was calculated to test the significance of the mediator. For the mediation analysis, the intervention was coded as PT and PTT = 1 and control group = 0.

### Local Research Ethical Review Requirement

The study was approved by an independent research ethics committee (Colworth Research Ethics Committee) in the South of England on October 21, 2009 (Multimedia Appendix 2). All research was conducted in accordance with the Helsinki Declaration [44].

## Results

### Participant Baseline Characteristics

At week 1, we contacted 1013 participants, of whom 96 did not meet the study’s inclusion criteria. We randomly allocated 858 into 1 of 3 study conditions, and 808 of them completed week 1. Table 1 shows participants’ baseline characteristics at week 1.

**Figure 2:**
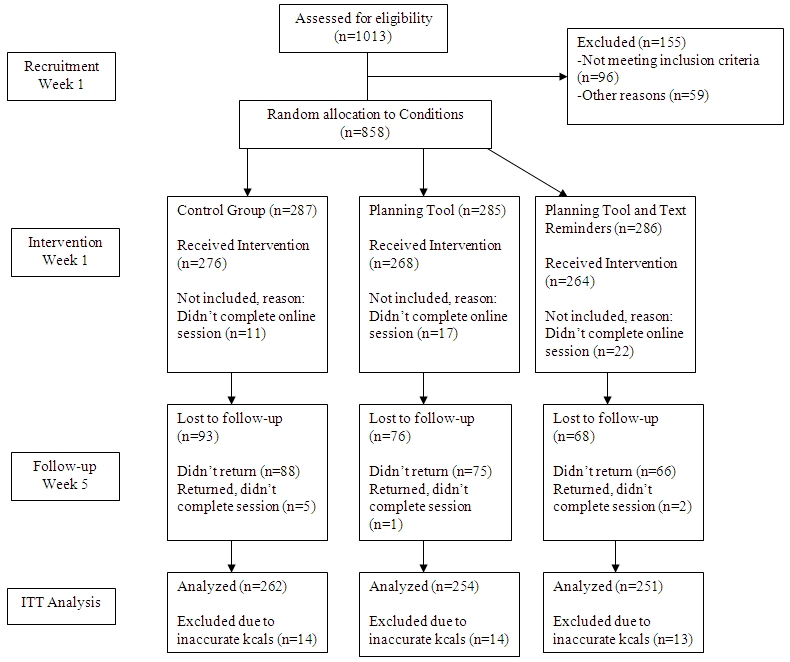
Flowchart of recruitment, intervention, and follow-up (ITT analysis = intention-to-treat analysis).

At week 5, a total of 571 participants revisited the website (70.7%). The number of participants completing each week is shown in Figure 2.

**Table 1:** Participant baseline characteristics (week 1)

	Overall (n = 808)	*F*_2__,805_ statistic	*P* value	Control group (n = 276)	Planning tool group (n = 268)	Planning tool and text reminders group (n = 264)
Age (years), mean (SD)^a^	46.0 (8.6)	0.23	.79	45.9 (8.4)	46.2 (8.6)	45.8 (8.7)
BMI^b^ (kg/m^2^), mean (SD)^a^	31.7 (5.9)	1.08	.34	31.3 (5.3)	31.7 (5.4)	32.1 (7.0)
% Saturated fat, mean (SD)^a^	15 (3.1)	0.93	.39	15 (2.9)	15 (3.2)	15 (3.1)
Smokers, n (%)^a^^,^^c^	24	0.09	.95	66/276 (23)	66/268 (24)	66/264 (23)

^a^ No significant differences found in participants’ baseline characteristics (*P* > .05).

^b^ Body mass index.

^c^ χ^2^ test statistic.

### Planning Tool

All participants allocated to the PT condition were able to formulate their plans online, and 85% completed 4 situation–solution pairs (83% PT and 87% PTT). The most frequently chosen situations were “If I’m getting a snack” (25%), “If I’m feeling hungry” (21%), “If I’m in a restaurant” (20%), and “If I feel like having a chocolate” (19%). The most frequently selected solutions included action plans such as “Then I will go for fruit” (18%), “Then I will go for chicken or fish” (15%), “Then I will have a smaller portion” (11%), and “Then I will find out about a lower fat option” (9%).

### Impact of the Intervention on Behavioral Outcomes

(1) The *2-item scale*: Participants in all 3 conditions reported a significant reduction in consumption of foods high in saturated fats between baseline and follow-up (Table 2). Analysis of covariance showed a significant difference between the conditions (*F*_2__,__571_ = 4.74, *P* = .009) with respect to self-reported saturated fat intake changes. Tukey–Kramer multiple comparisons indicated that this was due to participants in the PT and PTT conditions reporting a lower self-reported consumption of high-fat foods at week 5 than those in the control group.

(2) *F* *ood frequency questionnaire*: Participants in all conditions reported a significant reduction in percentage saturated fat intake between baseline and follow-up (Table 2) with no significant between-group differences (*P* = .23).

(3) *Portion s* *ize c* *hanges*: Participants in all conditions reported a significant reduction in their portion sizes for high saturated fat foods at week 5 (Table 2). There was a significant effect of condition on portion size changes (*F*_2__,569_ = 4.12, *P* = .017). Tukey–Kramer-adjusted multiple comparisons indicated a significant difference between participants in the PTT and control group (*P* = .02). Furthermore, a significant condition-by-gender interaction was found (*F*_3,569_ = 3.29, *P* = .02), with men in the PTT condition (mean 2.86, SE 0.084) reporting a greater reduction in their portion sizes than men in the control group (mean 3.27, SE 0.09; *P* = .003). This intervention effect was not found for women, who reported an average reduction of 2.92 (SE 0.01) in the PTT condition, 2.78 (SE 0.11) in PT, and 3.04 (SE 0.10) in the control group.

(4) *Other h* *ealth b* *ehavior* *s*: Analysis of covariance showed that there was no significant condition effect for any of the other health behaviors apart from “I ate a well-balanced diet” (*F*_2,570_ = 5.3, *P* = .005). Tukey–Kramer-adjusted multiple comparisons found a significant difference in eating a well-balanced diet between the PTT condition and the control group (*P* = .004).

**Table 2:** Saturated fat intake as measured by mean scores on a two-item scale, food frequency questionnaire, and portion size changes

	Control group (n = 276)			Planning tool (n = 268)			Planning tool and text reminders (n = 264)		
	Week 5	Week 5–1^a^	*t*_275_ (*P* value)	Week 5	Week 5–1^a^	*t*_2__68_ (*P* value)	Week 5	Week 5–1^a^	*t*_2__63_ (*P* value)
2-item scale	3.9	–1.1 (0.1)	<.001	3.6	–1.5 (0.1)	<.001	3.5	–1.2 (0.1)	<.001
Food frequency questionnaire	14.9	–0.7 (0.2)	<.001	14.5	–1.1 (0.2)	<.001	14.8	–0.8 (0.2)	<.001
Portion sizes	3.15	NA^b^	<.001	3.0	NA^b^	<.001	2.88	NA^b^	<.001

^a^ Mean change and standard errors after adjusting for baseline and other covariates.

^b^ Not applicable: portion size changes were assessed only at week 5, so mean change (week 5–1) was not calculated.

### Impact of the Intervention on Social Cognitive Variables

There was no significant condition effect on intention to reduce consumption of high-fat foods (*F*_2,570_ = 1.7, *P =* .18), maintenance self-efficacy (*F*_2,571_ = 0.7, *P* = .49), or recovery self-efficacy (*F*_2,571_ = 0.2, *P* = .86). There was a significant effect of condition on coping planning (*F*_2,571_ = 7.2, *P* < .001) but not on action planning (*P* = .16). For coping planning there was a significant difference between the PPT (mean 3.1; *P* < .001) and control group (mean 2.8), and PT (mean 3.0; *P* = .02) and control group, but not between the PTT and PT. Due to the above result, only the mediating properties of coping planning were tested. There was a significant relationship between the intervention (PTT and PT vs control group) and coping planning (beta = .23; 95% CI, .11–.35) and the intervention and the 2-item scale (beta = –.35; 95% CI, –.59 to –.11). Results from bootstrapping showed a significant mediation effect of coping planning (a*b = –.16; 95% CI, –.27 to –.08). We observed partial mediation since the effect of the intervention on self-reported saturated fat intake became nonsignificant and the b value was reduced but did not become zero (beta = –.20; 95% CI, –.42 to .02). At week 5, analysis of variance showed that there were no significant between-group differences in most of the feedback on the intervention items, apart from the worry item (*F*_2,570_ = 7.5, *P* < .001), with participants in the PTT condition (mean 3.21) reporting the information being less worrying than the control group (mean 3.9) (*P* < .001).

## Discussion

We found that participants completing a Web-based, fully automated planning tool alone or in combination with mobile text reminders self-reported a greater reduction in the consumption of high-fat foods than the control group. Participants in the PTT condition also reported higher levels of coping planning. Men in the PTT condition reported a greater reduction in the portion sizes of high-fat foods. Participants in the PT group without reminders did not change portion sizes more than those in the control group but showed higher levels of coping planning than the control group. We found no significant group differences in terms of percentage saturated fat intake, intentions, self-efficacy, or feedback on the intervention.

This study provides some support for the combined effects of implementation intentions plus text message reminders in reductions of high-fat foods (measured by the 2-item scale) and portion sizes. The two previous studies that combined implementation intentions with text messages found positive effects within the arena of physical activity and exercise behavior [18,19]. If implementation intentions operate by connecting the environmental cue with a desired response, then adding text messages may further strengthen either this connection or someone’s commitment to enact his or her plan [14].

Planning on its own showed a significant difference with the control group in relation to self-reported reductions in the 2-item scale but no significant differences for any of the other behaviors (eg, portion sizes, percentage saturated fat intake, and other health behaviors). Most of the evidence on the positive effects of implementation intentions comes from offline research studies. Forming plans online might be different from using a paper-and-pencil experiment. For example, in a Web-based study planning backfired so that people who did not form implementation intentions exercised significantly more than those who formed a Web-based implementation intention [13]. It is also the case that forming plans might not be effective, on their own, in some circumstances. For example, in a recent study [19], use of planning plus text reminders led to greater increases in exercise behavior than in the control group, yet there was no difference from control for those making plans with no text reminders.

Participants in all conditions reported a significant reduction in percentage saturated fat intake from the food frequency questionnaire and the 2-item scale. The existence of sufficiently high levels of motivation at the beginning of the study or a repeat measurement effect [45] might help explain this universal change and why, contrary to our expectations, there were no significant differences between groups for the percentage saturated fat measure. However, the 2-item scale was better able to differentiate between conditions than the food frequency questionnaire measure. Similar discrepancies in findings between percentage saturated fat intake and the 2-item scale have been reported before [9,12]. Both come with limitations, which we discuss later in this section.

The effects of the combined implementation intentions and text message condition on portion size changes were driven by male participants. Research on young females and males has suggested a difference in preferred communication style and use of technologies [46, 47]. Younger females (15–19 years old) tend to prefer in-depth conversations and to write longer messages with a more complex structure, while young males tend to be oriented toward simple one-thought messages and more task-oriented conversations [48, 49]. If reliable, these gender communication preferences would explain the differentiated impact of the text message reminders of plans, which was a rather task-oriented activity. An alternative explanation is that there was more scope for men to reduce their portion sizes. This could have led to a regression to the mean effect for men, so enhancing the apparent change in portion size. However, it has previously been reported that men tend to self-report consumption of bigger portions for solid, high-energy, and high-fat foods [50], main meals, and side dishes [51]. If this were true in our study then the larger reduction in portion size may have made it easier for men to reduce their portion sizes. The measurement of portion size change at a single point is a weakness of this study. Further studies are needed to compare changes in self-reported portion sizes by including pre- and postintervention measures.

We found no effect of the combined implementation intention and text reminders condition on other health areas (eg, weight loss and physical activity). In the current study, implementation intentions were paired up with plan reminders. Other research [20] has suggested that text reminders of people’s *goals* (eg, being healthier), but not plans, may activate related health behaviors (eg, dietary restriction) leading to other outcomes (eg, weight loss).

Contrary to our expectations and some previous research, there were no differences between the implementation intention conditions and the control group for social cognitive measures such as participants’ maintenance and recovery self-efficacy [12]. This might be because participants, who formed plans and encountered obstacles, needed further support to maintain their healthy eating and recover from lapses. Also, our intervention was not designed to increase self-efficacy beliefs. As suggested by the Health Action Process Approach model, strong self-efficacy beliefs could be the precursors of planning and behavioral change [4]. Indeed, self-efficacy has been found to predict whether people make plans about physical activity [52] and mediate the effects of planning on weight control [53]. Future interventions could first try boosting self-efficacy beliefs (through direct mastery experience or modeling) before the planning session.

Participants in the PTT and PT conditions chose mostly action rather than coping plans at week 1. Although there was no difference between conditions in the reported use of action plans at week 5, the participants in the PTT and PT conditions at week 5 reported using more coping planning than did the control group, and coping planning partially mediated the effects of the intervention on self-reported saturated fat intake. Hence, it seems that the use of coping plans was instrumental in driving changes in behavior. Previous research has suggested that action plans are more effective at the early stages of change, while coping plans are instrumental at later stages, and that a combined action and coping planning condition is better than a condition focusing solely on action plans [37,38]. In contrast to our results, a recent study on simultaneous use of action and coping plans found that they both mediated the intervention effects on fruit and vegetable intake [54]. Further studies are needed to more systematically explore the relative effectiveness of action and coping plans.

The impact of conditions on our measures of saturated fat intake changes was inconsistent, and this could be due to the limitations of the food frequency questionnaire and the 2-item scale. Underreporting of food consumption is a recurrent challenge for food frequency questionnaires and is most pronounced among overweight and obese people [55]. Also, food frequency questionnaires were initially designed to estimate individual intake relative to a population rather than to detect small changes in individual dietary intake [56], for which they might not be sufficiently sensitive. The original food frequency questionnaire calculation did not account for individual variation in portion sizes but instead assumed the average portion of the UK population [33]. When we considered portion sizes in this study, we found a significant difference between the PTT and the control group. On the other hand, self-reported items such as the 2-item scale were designed to detect differences between conditions in experimental studies [8,9]. However, some have claimed that reported changes are influenced by demand characteristics [57], with participants in more active conditions being more aware of study aims and so responding differently. Two previous studies counter the argument of demand characteristics by showing no difference between conditions for awareness of the study’s hypothesis or feelings of obligation to comply [9,13].

The current study has several advantages. It is based on a rather rigorous statistical analysis (ie, intention-to-treat analysis) and was conducted in a real-life setting. With a few exceptions most studies so far have been laboratory based and have examined effects of behavior in student populations [58,59]. This study also focused on overweight individuals, who are most likely to benefit from dietary interventions. Our planning tool is the first fully automated, interactive system to test Web-based if...then plans in the format of an interactive volitional help sheet, with the advantage of letting people choose the more personally relevant situations. Also, text messages were deployed with some flexibility by allowing participants to choose appropriate delivery times.

Limitations of the current study include the use of self-reports and the short-term follow-up of 4 weeks. It is essential that the combined effects of text messages and implementation intentions be tested over longer periods of time (ie, 6 months and 1 year) for sustained behavior change and that more objective measures of dietary behavior change be incorporated. Future research could also benefit by using ecological momentary assessment [60] so that text reminders of plans are sent at risky occasions, where cravings for high-fat foods occur.

### Conclusions

In conclusion, fully automated, Web-based planning tools and mobile technologies provide a good opportunity to promote large-scale dietary behavior change in overweight adults. In this exploratory study, a combined Web-based planning and text message condition was associated with some reductions in self-reported consumption of high saturated fat foods in comparison with the no-treatment control group.

## Multimedia Appendix 1

Research protocol (in lieu of prospective trial registration authors provide the research protocol).

## Multimedia Appendix 2

Ethics Committee Acceptance Letter.

## Abbreviations

PT: planning tool (condition)
PTT: planning tool and text messages (condition)
