# Supplementary material for: Exploratory Study of Web-Based Planning and Mobile Text Reminders in an Overweight Population
Source: J Med Internet Res. 2011 Dec 20;13(4):e118. doi: 10.2196/jmir.1773 (PMC3278104; doi:10.2196/jmir.1773)

**Final Protocol**

Study Title: **Use of Planning and SMS Reminders in the Promotion of a Healthy Diet**

Study Number: **UCR2009-1008**

Date: October 2009

## Contacts:

| |
|---|
| **SPONSOR:** Unilever Discover, Building 50, |
| Colworth Science Park, Sharnbrook, Bedfordshire |
| MK44 1LQ |
| **STUDY CO-ORDINATOR:** Natasha Soureti, Cyrena Tomlin, |
| Unilever Discover |
| 01234 248189, 01234-222148 |
| **PRINCIPAL INVESTIGATOR:** Bob Hurling, Unilever Discover, |
| 01234-222992 |
| |
| **TECHNICAL CONTACT:** Steve Baker, Tessella |
| Andy Hinton, Tessella |
| **MEDICAL CONTACT:** N/A |
| **IN CASE OF EMERGENCY: In case of a Serious Adverse Event (SAE) or similar emergency, one of the above must be contacted within one working day by telephone.** |
| **MONITOR:** N/A |

**Table of Contents**

**Part I: Summary**

Protocol Summary

Study Schematic

**Part II:**

**1. Study Overview**

1.1 Background information
1.2 Rationale for performing the study
1.3 Brief description of intervention
1.4 Summary of risks and benefits to the subjects

**2. Study Objectives**
2.1 Primary
2.2 Secondary

**3. Study Design**
3.1 Design and Rationale

**4. Study Population**
4.1 Total Number and Nature of Subjects
4.2 Subject Eligibility
4.3 Restrictions and Prohibitions

**5. Study Treatments**
5.1 Study Plan
5.2 Study Assessments
5.3 Study Duration
5.4 Dispensing and Accountability of Study Supplies
5.5 Continuation of Treatment

**6. Premature Withdrawal**
6.1 Withdrawal from the Study
6.2 Consequences of Missing Assessments, Withdrawals and Dismissals

**7. Investigational Events**
7.1 Endpoints
7.1.1 Primary
7.1.2 Secondary

**8. Experimental evaluations**
8.1 Biological Samples

**9. Safety**
9.1 Unilever Safety Clearance Requirements
9.2 Local Research Ethical Review Requirements
9.3 No Fault Compensation
9.4 Data Protection
9.5 COSHH
9.6 Head of Research
9.7 Microbiology

**10. Serious Adverse Events**

**11. Statistical Considerations**
11.1 Description of Statistical methods and interim analysis

11.2 Data Analysis and Plan of Efficacy
11.3 Sample size and power determination
11.4 Definition of Data and Analysis Population
11.5 Responsibilities for Statistical Analysis
11.6 Reporting of deviations of statistical plan

**12. References**

**Part III: General Considerations for Conducting a Protocol**

**1. Ethical Consideration**
1.1 Declaration of Helsinki
1.2 Good Clinical Practice

**2. Investigators Responsibilities**
2.1 Ethics
2.1.1 Declaration of Helsinki
2.1.2 Good Clinical Practice
2.1.3 Ethical Committee or Institutional Review Board
2.1.4 Informed Consent
2.1.5 Withdrawal of a Subject
2.1.6 Subject's Anonymity
2.2 Protocol Amendments
2.3 Case Report Form
2.4 Source Document Verification
2.5 Adverse Event
2.5.1 Adverse Event Definition
2.5.2 Unexpected Adverse Treatment Reaction
2.5.3 Causality
2.5.4 Adverse Event Severity Definition
2.5.5 Treatment & Follow-up of Adverse Events
2.6 Publication
2.7 Archiving
2.8 Audit

**3. Sponsor Responsibilities**
3.1 General Responsibilities
3.2 Monitoring and Contract Research Organisation Responsibilities
3.3 Quality Assurance & Quality Control
3.4 Archives
3.5 Final Report
3.6 Insurance

**Part IV: Appendices**

**Appendix 1: Study Schedule**
**Appendix 2: Example of the Online Informed Consent Form**
**Appendix 3: Questionnaires**
**Appendix 4: Planning**
**Appendix 5: Recruitment Agency Screening**
**Appendix 6: HAPA Model Diagram**
**Appendix 7: Participant Debrief**

## Appendix 8: Educational Information

---

### Part I: Protocol Summary

### PROTOCOL SUMMARY

| | |
|---|---|
| Title | **Planning and SMS Reminders in the Promotion of a Healthy Diet** |
| Location | Online |
| Study Objective (s) | The aim of the study is to investigate the role of Planning and SMS reminders in the promotion of a more balanced diet. |
| Study Design | Randomised Control Trial |
| Study Population Sample Size | 750 UK online users will be recruited, women and men between the ages of 30-60. |
| Intervention | An Internet based study. Volunteers will be allocated into one of three different conditions: a) Control Group, b) Planning (=Implementation Intentions) and c) Planning and Short Message Service (SMS) Reminders. At Time 1, all subjects will be asked to fill out a set of questionnaires and then will be allocated into one of these three conditions. Four weeks later, subjects will come back to the website and report on changes in healthy eating, saturated fat intake and various psychological determinants. |
| Study Endpoints | The primary endpoint is:<br>a) Self-reported saturated fat intake (Margetts et al , 1989) and portion size changes<br>The secondary endpoint is:<br>a) To assess changes in other health related behaviours<br>b) To test the intervention effects on social cognitive measures (e.g. recovery and maintenance self-efficacy, action and coping planning) measured by the Health Action Process Approach model. |
| Duration of Study | Time 1: 30mins<br>Time 2: 30mins |

---

### Part I: Study Schematic:

| **Phase 1** | **Phase 2** |
|---|---|
| 1) Subjects' Online Screening | 1) Filling out Questionnaires on:<br><br>a) Saturated Fat Intake<br><br>b) Social Cognitive Determinants<br><br>c) Feedback on Information Received |
| 2) Signing Online Consent Form | |
| 3) Filling out Questionnaires | |

| | |
|---|---|
| 4) Random allocation of subjects in one of three conditions:<br>a) Control Group<br>b) Planning<br>c) Planning and Short-Message Service (SMS) Reminders | |
| 6) Re-direction to Virtual Surveys' website | 2) Re-direction to Virtual Surveys' website<br><br>3) Payment of Subjects (stage 1: £5; stage 2: £10; payment received upon completion of Stage2) |

# 1. Study Overview

## 1.1 Background Information

A healthy diet, low in saturated fat and high in fibre, is a popular medical recommendation in helping people eat healthier. This type of recommendation would be particularly relevant to overweight or obese people, who may also want to lower or maintain a healthier cholesterol level. Most overweight people are likely to have good intentions to eat more healthily. However, good intentions are not always translated into action.

The emphasis of most current psychological models has been placed in identifying a number of factors that impact on intentions (motivational phase), leaving the translation between intentions into action less advanced (volitional phase). In this study, the focus is on the volitional phase of behaviour change. The Health Action Process Approach (HAPA) model (Schwarzer, 1999; Schwarzer et al, 2003; 2008) was chosen, since it addresses both the motivational phase and also the translation of motivation into behaviour. For a diagram of the HAPA model please see Appendix6.

In the HAPA model, a number of dimensions have been identified as playing an important role in the translation of intention into action namely a) action and coping planning and b) maintenance and recovery self-efficacy.

Maintenance self-efficacy is the confidence displayed by someone in dealing with barriers that arise once the behaviour has been initiated (Fuchs, 1997; Schwarzer & Fuchs, 1996; Luczcynska & Schwarzer, 2003). Recovery self-efficacy addresses the experience of failure and recovery from setbacks. Some people tend to trust more their competence at getting back on track after they've derailed and to reduce harm than others (Marlatt, 2002).

The inclusion of action and coping plans also known as implementation intentions are instrumental in helping to bridge the gap between intention and behaviour. Research on action plans is not new. A meta-analysis of 94 studies (Gollwitzer & Sheeran, 2006) showed that implementation intentions had a positive effect of medium to large magnitude on goal achievement. It also provided some evidence that implementation intentions can change dietary behaviours.

Action Planning is more than just an extension of intentions because it includes specific situational parameters. Evidence shows that people are less likely to forget their intentions when they specify the when, where and how manner (Gollwitzer & Sheeran, 2006). Coping planning is a different type of planning. The focus here is on the anticipation of barriers and the generation of alternative behaviours to overcome them. People think of possible scenarios that would hinder from their intended behaviour 'If I plan to run on Sunday and the weather doesn't permit it, then I will go swimming instead', 'If I want to have a steak (unhealthy, high fat option) when I go out, then I will go for smoked salmon instead'.

Fine tuning of planning is essential to goal attainment. Though previous research was more concerned with proving the value of using plans, more research is now needed on the mechanisms that make a plan effective. A number of different formats have been used in the past. In experiments, subjects have been asked to form a specific plan in as much detail as possible paying particular attention to the situation in which they will implement these plans. For example, '…Please write below when, where and how you will make an appointment…' (Sheeran & Orbell, 2000). Though important these statements fail to create a strong cue-response relationship. 'If…then' statements could be more beneficial than the more global goal intention instructions in two ways. Firstly, 'if' statements make the critical situation more accessible to the user and thus shifting detection to the situation once encountered in the environment. Secondly, the 'then' component of the plan creates a stronger link between the situational cue and the goal-directed response, automatically initiating action when the critical situation is encountered. For example, Oettingen et al (2000) compared the effects of forming a goal intention alone to a goal intention furnished with an 'if..then' format. Whereas in both cases a concrete situation was specified, subjects in the 'if..then' condition adhered more closely to the time specified in their plan. Chapman et al (2008) also found that 'if..then' statements increased significantly fruit and vegetable consumption compared with the more global goal intention format in a student population. The present study is one of the few ones to test 'If…Then…' statements online within the context of healthy eating.

**Short Message Service (SMS) Reminders**

It has been further argued that reminding people of their plans could enhance the impact of implementation intentions on behaviour (Gollwitzer et al, 1999; Sheeran et al, 2005). In a study by Prestwich et al (2008), subjects were randomly allocated into one of five conditions (Implementation Intentions and SMS reminders, Implementation Intentions, SMS reminders, and one of two Control Groups). In the follow-up four weeks later, results suggested a superiority of the combined condition in the frequency of exercise than the rest of the conditions, while neither the 'SMS reminders' or the 'implementation intention' groups were effective. In a later study, the same authors demonstrated that pairing Implementation Intentions with plan reminders or goal reminders increased the level of brisk walking relative to the control group. In addition, the goal reminder group lost more weight than the plan reminder group.

## 1.2 Rationale for Performing Study

The present study is an extension of a previous study conducted by Unilever Discover and approved by the Ethics Committee at the end of 2008. In this study we tested the effect of Heart-Age and the Planning tool on saturated fat intake. Heart-Age is a form of cardiovascular (CVD) risk communication. It is calculated as the age of a person with the same predicted risk but with all other risk factors levels in normal ranges. Heart-Age was found to be rather effective at helping people correctly perceive their CVD risk. Planning was more successful in changing behaviour e.g. lowering self-reported saturated fat intake. Effects were quite small in saturated fat changes and therefore it was necessary to start considering a) better measures of assessing behaviour changes and b) other ways of boosting the effects of implementation intentions on the outcome measures. This led to the present study, in which we combined planning with SMS reminders to test whether this could lead to stronger effects in behaviour than just planning on their own or nothing (Control Group).

## 1.3 Brief Description of Intervention

To the best of our knowledge there are no studies that have tested online the combined effects of planning with SMS reminders in a more complex behaviour such as dietary changes. In the present study, the aim was to test for differences on the effects of three different conditions on saturated fat intake and the purchase of products that contribute to lowering or helping maintain a healthy diet.

At **Time 1**, subjects will be invited to participate in the study by an online recruitment agency, which will also carry out the screening process. Once, subjects have been accepted in the study they will be randomly allocated into one of three conditions. In the most advanced condition, subjects will be asked to form implementation intentions and be reminded of their plans by SMS reminders over the course of the two weeks. In the second condition, they will be asked to form plans on how to eat healthier over the course of two weeks. All conditions will be asked to fill out questionnaires on: a) a self-reported dietary intake questionnaire (Margetts et al, 1989) and b) a self-reported questionnaire on various social cognitive variables (e.g. intentions, self-efficacy). The Control Group (CG) will be only asked to fill out these questionnaires at this stage. All three groups will receive educational information on the importance of a healthy diet low in saturated fat and the association between high cholesterol and being overweight. A coupon of a cholesterol lowering product, with 50p off the face value, will be posted to all participants as a reward for successfully completing baseline assessments.

At **Time 2 (4 weeks later)**, subjects will be invited to fill out again the same dietary measures of food intake and an extension of the psychological determinants' questionnaire. Study subjects will be rewarded for their participation in the study (£15). This will be upon completion of study phase 2.

## 1.4 Summary of risks and benefits to the subjects

### Benefits

The main benefit to the subjects:

a) Starting a two-way interaction is crucial if we want subjects to start taking personal responsibility for their health. This study will attempt to engage them in the use of a planning tool to start making healthier eating choices and in the use of products and services that could help them maintain a healthier lifestyle.

### Risks

There is no perceived risk from engagement of participants with the planning tool or SMS reminders.

## 2. Study Objectives

### 2.1 Primary Objectives

**Objectives:**

The primary aim of the present study is to test the effects of Planning and SMS Reminders on saturated fat intake and purchase of a healthy product over a period of two weeks.

To assess the above, three questionnaires will be issued:

1. Margetts et al (1989) food frequency questionnaire
2. A two item self-reported saturated fat intake measure
3. Self-reported changes in portion sizes

### 2.2 Secondary Objectives

The secondary objectives of this study are to evaluate:

1. The effects of planning and SMS reminders on intentions, maintenance and recovery self-efficacy, action and coping planning and feedback of the intervention items.
2. The mediation effects of various constructs of the health action process approach (HAPA) Model (e.g. planning) on behaviour.

## 3. Study Design

### 3.1. Design and Rationale

This is a randomised, stratified between-groups study designed to assess the difference in saturated fat intake and coupon up-take between the three different experimental conditions; a) Planning and SMS reminders, b) Planning and c) Control Group. Subjects will be randomly allocated to one of these conditions and their responses will be measured by self-reported questionnaires.

Allocation to the subjects in the conditions will be stratified to balance by age group (30-45 years or 46-60 years), and gender. To help minimise any other imbalance effects obese ($BMI \geq 30$) and non-obese participants ($BMI \geq 25$), smokers and non-smokers will be also evenly allocated across the different cells.

## 4. Study Population

### 4.1 Total Number and Nature of Subjects

We will over-recruit at Time 1 to be able to take into account any drop-outs likely to occur over the study period. Seven hundred and fifty participants, who are motivated to make changes in their diet for a healthier cholesterol level will be recruited. Motivated subjects have been selected, since plans might sometimes fail to change behaviour if the individual does not hold sufficiently positive intentions to perform the planned behaviour (Prestwich et al, 2003; Sheeran et al, 2005).

Subjects will be recruited from both genders, be 30-60 years old with a BMI≥25. Gender and age groups (30-45, 46-60 year olds) will be evenly distributed in the different cells. BMI will be split into two categories: a) overweight 25≥BMI≤29.9 and b) obese BMI≥30.
Overweight and obese subjects have been selected, since they are expected to take into consideration their weight when making health-related judgements. They are more likely to consider themselves as being at higher risk than people of average weight (Renner et al, 2000) and therefore more likely to want to make dietary changes.

### 4.2 Subject Eligibility

Subjects will be screened by use of an online questionnaire assessing weight/height, age, gender, pre-existing cardiovascular disease and cancer.

**Exclusion Criteria:**

- BMI ≤ 24.9 (Body Mass Index)
- <30 years old
- >60 years old
- Pregnant women
- Diagnosed with cancer
- Diagnosed with an eating disorder
- Diagnosed with a heart-condition (heart-attack or angina)
- Any other chronic disease of the major organs (e.g. kidney failure)
- Not willing to sign online consent form
- Not literate in use of computer and the internet
- Not being able to print at home or at work
- Not having their own mobile phone
- Not capable of opening delivered SMS messages
- Not willing to receive SMS messages over the duration of the study

**Inclusion Criteria**

- BMI ≥25
- 30-60 years old
- Subjects of either sex can take part
- Not diagnosed with a heart-condition (heart-attack or angina)
- Not diagnosed with cancer
- Not diagnosed with an eating disorder
- Willing to sign the Informed Consent Form
- Computer and internet literate
- Being able to print at home or at work
- Having their own mobile phone
- Being capable of opening delivered SMS messages
- Be willing to receive SMS messages over the duration of the study

### 4.3 Restrictions and Prohibitions

There will be no restrictions or prohibitions placed once subjects have entered the main study-phase.

## 5. Study Treatments

### 5.1 Study Plan

The flow of the different study phases is seen in Figure 1 below.

Figure 1 – Study Flow

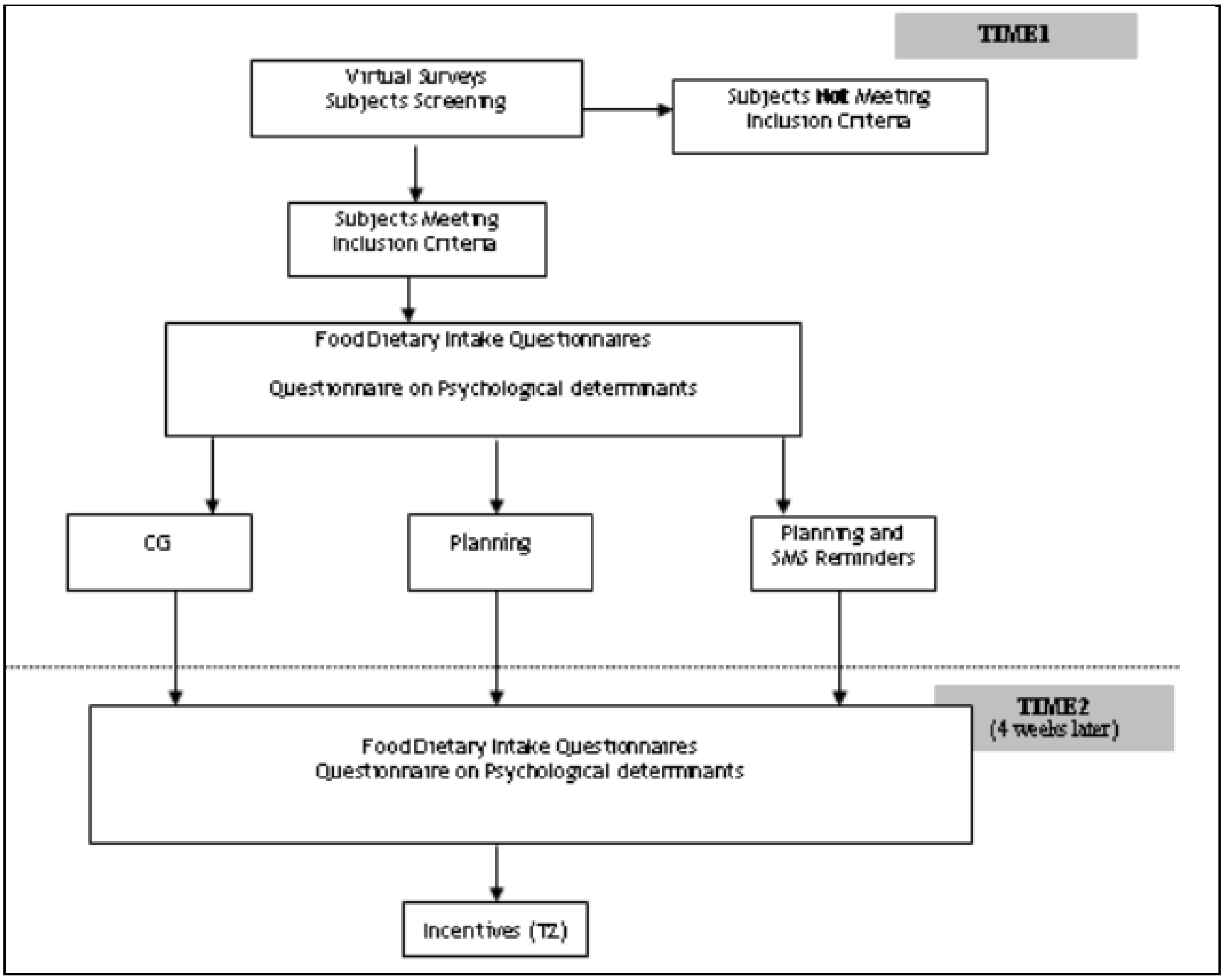


## Phase 1

a) **Recruitment and Screening**

Recruitment will be conducted by Virtual Surveys (http://www.virtualsurveys.com/s), an online recruitment agency, to cost effectively reach a specific target sample within the UK population. Virtual Surveys have their own online panel and a structured script will be used to screen individuals and exclude any people who do not meet the inclusion criteria (Appendix 5). Once subjects meet the inclusion criteria, they will be acquainted with the study procedures and asked to read an online informed consent form. They will be requested to click on a number of check boxes to make sure they have read and understood the information before they proceed to the online questionnaires. There will be two types of questionnaires at this phase: a) food frequency questionnaire and b) questionnaire focusing on social-cognitive determinants.

**b) Intervention**

In the main study phase, subjects will be randomly allocated into one of three different conditions: a) Control Group, b) Planning and c) Planning and SMS Reminders. Subjects in the experimental conditions will be first provided with information on a healthy diet, the importance of good fats in a person's diet and the link between cholesterol, diet and overweight.

Subjects in the planning condition will then identify a list of situations where they would like to change their dietary intake. They will then be asked to match these situations with a list of behaviours. They would be requested to choose pairs of situations and behaviours that they want

to change rather than ones that they are already doing (Appendix 4). The list of behaviours is based on the process of change model of Prochaska (1979). It includes strategies such as counterconditioning, stimulus control and use of helping relationships. A number of more nutritionally based behaviours have also been created with the help of an expert nutritionist and information from accredited sources such as the Food Standard's Agency 'eat well, be well' site (http://www.eatwell.gov.uk/). Once, they have made their selections they will be given the option to go back to improve their plan or revise their situation-solutions.

Subjects in the SMS reminders and planning condition will also follow the same process to choose their situation-solution pairs and then be provided with SMS reminders of these pairs over the course of the 4 weeks. Two SMS reminders will be sent per week. Subjects will be able to choose the time of the day they would like to receive the SMS reminder. Subjects will be also informed that use of their mobile number for the SMS reminders will be made only during the duration of the study and they will not be contacted any further upon completion of the study.

All subjects will be invited to answer a number of questions on their perceptions of the information received, intentions to make lifestyle changes, feelings of self-efficacy. Subjects in the control group will be told that they will be put on a waiting list and will receive further information in four weeks time.

**Phase 2 – Study End**

In the last phase, subjects will come back to the online study site to fill out a number of questionnaires. Subjects in the control group will at this stage be provided with the same educational information that the rest of the subjects received at Time1.

Upon completion of the study, all subjects will be thanked and debriefed (Appendix 7) and directed back to the Virtual Surveys site to receive payment (£15) for participation in the study.

### 5.2 Study Assessments

Subjects will complete a number of questionnaires.

**Primary Outcome**

Saturated Fat Intake

a) Self-reported amount of saturated fat intake will be measured by Margetts et al (1989) 63 item food frequency questionnaire and a two-item self-reported measure. The Margetts, Cade & Osmond (1989) questionnaire is a validated self-report index of food. The frequency of consumption of 63 common foods over the previous month using a 6-point-scale is assessed (two or more times per day; three to five times per week; one to three times per month; rarely or never). The scale has good test-retest reliability (r=.61; Armitage & Conner, 1999). In this study a column on reduction on portion sizes will be added and assessed at Time2.

b) Saturated fat intake will be also measured by a two item self perception questionnaire.

c) Portion Size Changes

Portion size changes in the consumption of 11 high fat items (e.g. meat dishes, whole milk, bacon, cheese ordinary, chocolate, crisps) will be assessed. These items accounted for the highest reported saturated fat intake in the FFQ from a previous study. Participants will be asked to report changes in their portion sizes on a 7 point Likert scale (from 'a lot less' to 'a lot more').

**Secondary Outcomes**

Other Health Behaviours

Other health behaviours will be measured by 6 items on a 7 point Likert scale (from 'a lot less' to 'a lot more'). Participants will be asked to report changes in other health areas namely alcohol, use of cholesterol lowering products, weight changes, smoking, physical activity and eating a well balanced diet.

Self-Efficacy/ Planning/Intentions

There will be a second questionnaire that will cover a range of psychological determinants. The maintenance and recovery self-efficacy, action and coping planning, intentions measures have been adapted from Renner & Schwarzer's (2007) and Schwarzer's (2008) publication on assessment of the Health Action Process Approach Model (HAPA) and from Sienhotta et al.'s (2005) paper on physical activity.

Reactions to the Intervention

Subjects' opinions of the intervention were adapted from Bourdeaudhuij & Brug (2000), and Brug et al (1998). Two extra items were included on emotional reactions to the information presented ('I felt that the information…..was worrying', …'a wake-up call').

### 5.3 Study Duration

This study requires two online visits to a web site. The same subjects will not be contacted in the future to fill out any other information. The whole process, including screening, main study phase, and follow-up should not last more than 1 hour.

### 5.4 Dispensing and Accountability of Study Supplies

N/A

### 5.5 Continuation of Treatment

The study link will be only designed to be used during the study duration. Upon completion of the study, those subjects that are happy will be provided with a link to the Flora website http://www.florahearts.co.uk/Consumer/Home.aspx and will be given the opportunity to test their Heart-Ages and sign up to a free Health Plan. Subjects will be provided with instructions by Virtual Surveys on how to get the free health plan once they visit the website.

## 6. Premature Withdrawal

### 6.1 Withdrawal from the Study

If a subject meets the inclusion criteria but decides to withdraw mid-way through the study then no data will be uploaded in the system and a request will be made to virtual surveys for this person to be replaced by another subject with the same characteristics.

### 6.2 Consequences of Missing Assessments

There are two assessment points in this study. Virtual Survey has suggested a 30% drop out from one study phase into the next. To account for this, more than 725 subjects will be recruited to end up with roughly 500 subjects at Time2.

## 7. Investigational Events

### 7.1 Endpoints

#### 7.1.1 Primary

The primary endpoint is:

a) Self-Reported Saturated Fat Intake
b) Portion Size Changes

#### 7.1.2 Secondary

The secondary endpoints are:

a) Other health behaviours
b) Intention to reduce saturated fat intake
c) Maintenance and recovery self-efficacy
d) Action and coping planning

e) Reaction to the intervention

## 8. Experimental evaluations

### 8.1 Biological Samples

No biological samples are being collected in this study and there are no invasive measures.

## 9. Safety

Subjects will be recruited only when the appropriate approvals have been granted.

### 9.1. Unilever Safety Clearance Requirements

N/A

### 9.2. Local Research Ethical Review Requirement

The authorised protocol and other appropriate documentation will be subject to the approval of Colworth Research Ethics Committee. The study will not proceed without the written approval of the Ethics Committee and any recommendations for change or additions will be discussed and necessary changes made. Finally the Research Ethics Committee will be sent a summary of the study findings for their information.

### 9.3. No Fault compensation

In the unlikely event of any subject suffering any significant deterioration in health or wellbeing arising as a result of taking part in this study, Unilever has undertaken to compensate them without having to prove that anyone involved in this study has been negligent. Any dispute will be referred to an arbitrator.

### 9.4. Data Protection

Information relating to this study will be kept confidential and the subjects' right to check personal data will be fully protected in accordance with the Data Protection Act 1998. Use of Case Record Forms is not applicable in this study since all information will be gathered via an online system. Each subject will be allocated with a unique ID at the recruitment stage (by Virtual Surveys) which will remain the same until the end of the study. Subjects will be responsible for entering their data on the system and no personal details such as (first name, last name and email addresses will be asked of the subjects). Subjects will be though required to fill out some personal health information, gender, date of birth, mobile number to receive feedback at the end of the study. To secure any personal information, data such as mobile numbers will be stored by a third party to Unilever into a different database with a higher level of security. This data will be deleted upon participants' study completion. Participants will also be informed that their mobile numbers will be only used during the duration of the study and will be deleted upon completion of the study.

The subject may choose not to provide certain information, but this may mean they will not be able to complete the study and their data will be replaced by new participant data. The site will not contact the user and Unilever Discover will not knowingly sell, rent, or trade the personal information collected.

All web-based interaction data (apart from mobile numbers) will be stored for a period of five years in a secure central database hosted by Unilever. Only a few project members will have access to this folder. Data files on the mainframe will be backed-up automatically. All data files will be backed-up and stored in a secure location.

### 9.5 COSHH

N/A

### 9.6 Head of Research

Once all of the study approvals have been collected, the Corporate Clearance form will be signed off by the Head of Corporate Research.

### 9.7 Microbiology
N/A

## 10. Serious Adverse Events

Any adverse event that is considered SERIOUS must be reported within one working day by the investigator to the sponsor/study co-ordinator (for definition see Protocol part III; section 2.5 "Adverse Event").

## 11. Statistical Considerations

### 11.1 Description of Statistical methods and interim analysis

This is a randomised, stratified, between-groups study with the factors 'Planning' and 'SMS Reminders'. The primary endpoint is the change in saturated fat and portion size changes in participants allocated to the three different experimental conditions (Planning, Planning and SMS Reminders and Control Group). Participants will be randomly allocated to one of these three conditions, stratifying for age group (30-45 years or 46-60 years) and gender. Self-reported portion size changes will be measured at a single point at the end of the study. Participants' saturated fat intake will be measured at baseline and upon completion of the study (2 weeks after enrolment). The statistical analysis will compare the efficacy of the three treatment groups via the change in saturated fat intake between the baseline and final diet assessments and change in portion sizes at the end of the study. There will be no interim analyses.

### 11.2 Data Analysis and Plan of Efficacy

A full statistical analysis plan will be formulated in collaboration with Peter Murray from the Unilever Statistics Group, Colworth Park. The effect of the design factors will be determined by comparing the proportion of subjects who have reduced their self-perceived saturated fat intake. Analysis of variance with baseline covariates will be conducted to analyse the behavioural (TIS, FFQ, portion sizes changes, other health behaviours) and social-cognitive measures (intentions, self-efficacy, planning, feedback). Baseline self-reported saturated fat intake will be included as a covariate for the analysis of the primary outcome measures. The stratification variables will be included in the models as will other covariates found significant at the 0.05 level. Comparisons between treatments will all be reported using two-sided significance levels (i.e. making no prior assumption about which treatment group will be most effective). Separate analysis of the effects of modifiers and mediators will be conducted in order to gain a better insight as to who benefits from the intervention.

### 11.3 Sample size and power determination

The primary endpoint of self-perceived SAFA intake reduction will be assessed using analysis of variance with baseline covariates. Data from a previous study indicates that around 180 respondents should be sufficient to give power 0.8 of seeing a significant difference (at the two-sided 0.05 level) between the control and planning groups for this endpoint. No prior data exists for the portion size change measure.

### 11.4 Definition of Data and Analysis Population

Data will be recorded by subjects, captured via the on-line system and will be held in compliance with the UK Data Protection Act. Each subject will be identified by a unique ID number assigned to them.
The original data will be stored in the computer (but no hard-copies will be kept since this is an online study) and will be filed at Unilever for a minimum of five years from completion of the

study.

### 11.5 Responsibilities for Statistical Analysis

All statistical analyses will be performed by the Unilever Statistics Group, Colworth Park

### 11.6 Reporting of deviations of statistical plan

Any deviations of the statistical plan will be reported in the analysis.

## 12. REFERENCES


Bourdeaudhuij, I.D., & Brug, J. (2000). Tailoring dietary feedback to reduce fat intake: an intervention at the family level. *Health Education Research: Theory and Practice,* 15 (4), 449-462.

Brug, J., Glanz, K., Assena, P., Kok, G., Breukelen, G.J.P., (1998). The impact of computer-tailored feedback and iterative feedback on fat, fruit and vegetable intake, *Health Education and Behaviour,* 25 (4), 517-531.

Chapman, J., Armitage, C.J., & Norman P. (2008). Comparing implementation intention interventions in relation to young adults' intake of fruit and vegetable. *Psychology and Health,* 1-16

Costa, P. T., Jr., &McCrae, R. R. (1992). *Revised NEO Personality Inventory (NEO-PI-R) and NEO Five- Factor Inventory (NEO-FFI) professional manual.* Odessa, FL: Psychological Assessment Resources.

Franks P (2009). Five factor model personality factors moderated the effects of an intervention to enhance chronic disease management self-efficacy. *British Journal of Health Psychology,* 14, 473-87.

Gollwitzer, P. M & Sheeran P (2006). Implementation intentions and goal achievement: a meta-analysis of effects and processes. *Advances in Experimental Social Psychology,* 38, 69-111.

Gosling, S. D., Rentfrow, P. J., & Swann, W. B., Jr. (2003). A Very Brief Measure of the Big Five Personality Domains. *Journal of Research in Personality, 37, 504-528.*

John, O. P., & Srivastava, S. (1999). The Big Five trait taxonomy: History, measurement, and theoretical perspectives. In L. A. Pervin, & O. P. John (Eds.), *Handbook of personality: Theory and research* (pp.102–138). New York: Guilford Press.

Luszczynska, A& Schwarzer, R (2003). Planning an self-efficacy in the adoption and maintenance of breast self-examination: a longitudinal study on self-regulation cognitions, *Psychology and Health,* 18 (1), 93-108.

Margetts, B.M., Cade, J.E., & Osmond, C. (1989). Comparison of a food frequency questionnaire with a diet record, *International Journal of Epidemiology,* 18, 868-873.

Marlatt, G.A. (2002). *Harm reduction: Pragmatic strategies for managing high-risk behaviors.* New York: Guildford

Oettingen , G., Honig, G., Gollwitzer, P. M. (2000). Effective self-regulation of goal attainment. *International Journal of Educational Research*, 33, 705-732.

Orbell, S & Sheeran, P (2000). Motivational and volitional processes in action initiation: a field study of the role of implementation intentions. *Journal of Applied Social Psychology,* 30, 780-797.

Prestwich, A.J., Lawton, R.J., Conner, M.T. (2003). The use of implementation intentions and the decision balance sheet in promoting exercise behaviour. *Psychology and Health,* 18, 707-721.

Prestwich, A., Perugini, M., & Hurling R. (2008). Can the effects of implementation intentions on exercise be enhanced using text messages? *Psychology and Health,* 1-11.

Prochaska, J. O. (1979). Systems of psychotherapy: A transtheoretical analysis. Homewood. IL: Dorsey Press.

Robins, R. W., Trzesniewski, K. H., Tracy, J. L., Gosling, S. D., & Potter, J. (2002). Self-esteem across the lifespan. *Psychology and Aging,* 17, 423–434.

Schwarzer, R (1999). Self-regulatory processes in the adoption and maintenance of health behaviours. The role of optimism, goals and threats. *Journal of Health Psychology*, 4, 115-127.

Schwarzer, R., Sniehotta, F. F., Lippkie, S., Luszynska, A., Scholz U., Schuz B., Wegner M., Ziegelman, J (2003). On the assessment and analysis of variables in the health action process approach: conducting an investigation. In http://web.fu-berlin.de/gesund/hapa_web.pdf

Schwarzer, R .(2008). Modelling health behaviour change: how to predict and modify the adoption and maintenance of health behaviours, *Applied Psychology: An International Review,* 57 (1), 1-29.

Sheeran., P., Webb, T.L., & Gollwitzer, P.M. (2005). The interplay between goal intentions and implementation intentions, *Personality and Social Psychology Bulletin,* 31, 87-98.

Sniehotta, F. F., Scholz, U., & Schwarzer, R. (2005). Bridging the intention-behaviour gap: Planning, self-efficacy, and action control in the adoption and maintenance of physical exercise, *Psychology and Health*, 2, 143-160.

Webb TL, Christian J, and Armitage CJ (2007). "Helping students turn up for class: does personality moderate the effectiveness of an implementation intention intervention?" *Learning and Individual Differences,* 17, 316-27.

## Part III: General Considerations for Conducting a Protocol

### 1. Ethical Considerations

#### 1.1 Declaration of Helsinki

The current revision of the Declaration of Helsinki is the accepted basis for clinical study ethics, and must be fully followed and respected by all engaged in research on human beings. Any exceptions must be justified and stated in the protocol. Independent assurance that subjects are protected can only be provided by an ethics committee/institutional review board and freely obtained informed consent.

#### 1.2 Good Clinical Practice

Good clinical practice is a standard for clinical studies, which encompasses the design, conduct, monitoring, termination, audit, analyses, reporting and documentation of the studies. It ensures the studies are ethically justified and scientifically sound, and that the clinical properties of the diagnostic/therapeutic/prophylactic product under investigation are properly documented. Refer to section 2.1.2 for further guidance.

## 2. Investigators Responsibilities

It is the responsibility of the investigator(s) to conduct the study according to the protocol and to ensure that (s) he has the subject availability to conduct the study within the period defined in the study protocol.

### 2.1 Ethics

#### 2.1.1 Declaration of Helsinki

It is the responsibility of the investigator(s) to ensure that the study is conducted in full conformance with the principles of the current Revised Version (1989) of the Declaration of Helsinki.

#### 2.1.2 Good Clinical Practice

It is the responsibility of the investigator(s) to ensure that the study is performed in accordance with Unilever's standards for the conduct of Human Trials and according to all local laws and regulations concerning Human studies. It is observed that due to the nature of Unilever human trials these do not qualify as Clinical Trials and as such, are not obliged by law to follow EU Directive 2001/20/EC (Clinical Trials Directive) or EU Directive 2005/28/EC (GCP Directive). However, Unilever Human Trials where possible operate to the spirit of these Directives in order to ensure best practice.

#### 2.1.3 Ethics Committee or Institutional Review Board

It is the responsibility of the investigator(s) to submit a copy of the protocol and consent form to an ethics committee/institutional review board in order to obtain independent approval to conduct the study. Ethics committee/institutional review board approval must be obtained before the study is started. The approval of the ethics committee/institutional review board must be sent in writing, to the investigator(s). The Ethics Committee approval letter must mention the Ethics Committee members and their function.

#### 2.1.4 Informed Consent

It is normally the responsibility of the investigator(s) to obtain informed consent from each subject participating in the study, after explanation of the aims, methods, benefits and potential hazards of the study. Since this study is solely conducted online the aims, benefits and potential harms to the subjects will be explained online (Appendix 2). The consent must and will be obtained before any study-specific procedures are performed.

It must be made completely and unambiguously clear to each subject that they are free to refuse to participate in the study, or that they can withdraw their consent at any time and for any reason, without incurring any penalty or withholding of treatment on the part of the investigator. The online action required for informed consent must be recorded and kept on file by the investigator(s).

#### 2.1.5 Withdrawal of a Subject

N/A

### 2.1.6 Subject's Anonymity

The investigator(s) must ensure that the subject's anonymity will be maintained. On all documents, subjects must be identified only by an identification code and not by their names. To secure subjects' anonymity two third parties to Unilever will be involved.Virtual Surveys (online recruitment agency) will keep a separate confidential enrolment log that matches identifying codes with the subject's names and addresses. Tessella (IT development agency) will keep hold of subjects' mobile numbers to send the SMS Reminders and will delete the data upon completion of the study.

### 2.2 Protocol Amendment(s)

Any modification to the agreed protocol must be approved in writing by the sponsor/ study co-ordinator and the investigator(s). If alterations have to be made to the protocol after Ethical approval has been given, then the amendments will be submitted for their approval and not implemented until approval is received.

### 2.3 Case Report Form

N/A

### 2.4 Source Document Verification

N/A

### 2.5 Adverse Event

In the current study no treatment or direct intervention is offered and no medical tests are performed. Therefore, it is considered unlikely that subjects will experience adverse event as a result of participation in the study.

### 2.5.1 Adverse Event Definition

Any untoward medical occurrence in a study subject or administered a treatment and which does not necessarily have to have a causal relationship with the treatment. An adverse event can therefore be any unfavourable and unintended sign (including an abnormal laboratory finding), symptom, or disease temporally associated with the use of a treatment, whether or not considered related to the treatment. Pre-existing conditions that worsen during a study are to be reported as adverse events. They can become Serious Adverse Events if they fulfil one of the seriousness criteria described below.

**SERIOUS ADVERSE EVENTS**

Any adverse event that fulfils at least one of the following criteria:

- It is Fatal (results in death) (***note:*** death is an outcome, not an event).
- It is Life-Threatening (***note:*** the term "life-threatening" refers to an event in which the subject was at risk of death at the time of the event; it does not refer to an event which could hypothetically have caused death had it been more severe).
- It requires subject hospitalisation or prolongation of existing hospitalisation (***note***: "inpatient hospitalisation" refers to an unplanned, overnight hospitalisation).
- It results in persistent or significant disability/incapacity.
- It is a congenital abnormality/birth defect.
- It is medically significant or requires intervention to prevent one or other of the outcomes listed above.

Any adverse event that is considered SERIOUS must be reported. Since no treatment is offered to subjects and no medical tests conducted there is no such occurrence expected. Please see section

2.5.1

### 2.5.2 Unexpected Adverse Treatment Reaction

An adverse reaction, the nature or severity of which is not consistent with the expected risks of participating in the study.

### 2.5.3 Causality

Causality can be one of three possibilities:

- "NO" (definitely not treatment related)
- "YES" (remotely, possibly, probably or definitely treatment-related)
- "UNKNOWN"

All adverse events judged by either the investigator or the sponsor as being definitely not "NON TREATMENT-RELATED" qualify as Adverse Treatment Reactions.

### 2.5.4 Adverse Event Severity - Definition

The severity/intensity of adverse events can be graded either on a three-point W.H.O. scale (*See W.H.O. Handbook for Reporting Results of Cancer Treatment)* e.g.

- **Mild** or *Grade 1*: discomfort noted, but no disruption to normal daily activities.
- **Moderate** or *Grade 2*: discomfort sufficient to reduce or affect normal daily activities.
- **Severe** or *Grade 3*: Inability to work or perform normal daily activities.

### 2.5.5 Treatment and Follow-up of Adverse Events

N/A

### 2.6 Publication

Information relating to the study will be kept confidential and publications will be in a form with which the panellist cannot be identified.

### 2.7 Archiving

It is the responsibility of the investigator(s) to maintain adequate clinical study records. Copies of all clinical study material must be archived in the computer electronically (no hard-copies will be kept since this is an online study) for a minimum of five years from completion of the study (or more as legally required). All paper and electronic documents must be archived in a secure place and treated as confidential material.

### 2.8 Audit

The investigator agrees to comply with the sponsor and regulatory authority requirements regarding the auditing of the study.

## 3. Sponsor Responsibilities

### 3.1 General Responsibilities

The project manager/study co-ordinator, the investigator(s) and the study statistician will provide a final draft **protocol**. After mutual agreement, all parties sign the study protocol and submit the protocol to Ethics Committee.

The sponsor/study co-ordinator provides the investigator(s) with **sufficient material** and support

to permit the investigator(s) to conduct the study according to the agreed protocol. The sponsor/study co-ordinator reserves the rights to **terminate the study prematurely** for persistent protocol violations, or any other valid and ethical reasons. Should this be the case, the necessary procedures will be arranged after review and consultation by both parties to ensure protection of the subject's interests.

The sponsor provides treatment and/or **compensation** in the case of an injury to, or the death of, a subject, as a result of his/her participation in a study sponsored by them. The sponsor also **insures** the investigator(s) against, or indemnifies them for, losses resulting from liability for such injuries or deaths.

### 3.2 Monitoring and Contract Research Organisation Responsibilities

N/A

### 3.3 Quality Assurance and Quality Control

All material used in studies are subjected to **quality control**. Quality assurance audits may be performed by the sponsor or any health authority during the course of the study or at study completion.

### 3.4 Archives

The protocol, approvals and all other essential documents related to the study must be archived, including certificates that satisfactory audit and inspection procedures have been carried out. All documents must be archived in a secure place for 5 years and treated as confidential material.

### 3.5 Final report

A summary will be issued within four weeks of study completion and a final report within 4 weeks of sponsor's comments. This report should include number of subjects enrolled, number of subjects dropped/withdrawn, deviations, amendments, statistical analysis and general or specific comments from the investigator. Additionally, the contract laboratory will provide the sponsor/study co-ordinator with the electronic data.

### 3.6 Insurance

In the unlikely event of any subject suffering any significant deterioration in health or wellbeing arising as a result of taking part in this study, Unilever has undertaken to compensate them without having to prove that anyone involved in this study has been negligent. Any dispute will be referred to an arbitrator.

---

## PART IV: Appendices

**APPENDIX 1 - Study Schedule**

**APPENDIX 2 - Example of Informed Consent Form**

**APPENDIX 3 – Questionnaires**
**APPENDIX 4 – Planning**
**APPENDIX 5– Recruitment Agency Screening**

**APPENDIX 6- HAPA Model Diagram**

**APPENDIX 7 – Participant Debrief**

**APPENDIX 8 – Educational Information**

## APPENDIX 1 - Study Schedule

| **Phases** | **Processes** |
|---|---|
| **Time 1:**<br><br>Recruitment/Screening<br>(Virtual Surveys) | -Screening questionnaire (height, weight, health status, age, gender, cholesterol and healthy eating motivation).<br><br>-Online signing of consent form. |

| | |
|---|---|
| Conduction of Intervention | -Allocation to different experimental conditions.<br><br>-Questionnaire assessing subject's intentions, saturated fat intake, self-efficacy, planning etc. |
| **Duration** | 30mins |
| **Time 2**<br><br>Post-Study Assessment<br><br>Payment of participants | -Follow-up questionnaire assessing subject's reactions, self-efficacy, planning, saturated fat intake, intentions.<br><br>-Subjects directed back to Virtual Surveys to be paid |
| **Duration** | 30mins |

## APPENDIX 2 - Example of Informed Consent Form

**Study title: A healthy diet**

You are being invited to take part in a research study. Before you decide it is important for you to understand why the research is being done and what it will involve. Take time to decide whether or not you wish to take part.

**What is the purpose of the study?**

The aim of this study is to assess the healthiness of your diet and help you maintain a balanced diet for a healthy cholesterol level.

**Do I have to take part?**
It is up to you to decide whether or not to take part. If you do decide to take part you will be asked to sign an online consent form. If you decide to take part you are still free to withdraw **at any time** and without giving a

reason. If you withdraw at any time before the completion of a study phase then you won't get reimbursed for your time.

**What will happen to me if I take part?**

You will be asked a number of health-related questions some of which you might have answered before. You will be provided with information on the link between fats, a balanced diet, and be provided with advice on how to achieve a healthier diet. You might be asked to provide your mobile number. Once you have been through the consent form, there are two study phases. Both will last about 30minutes.

**What are the possible benefits of taking part?**

The most likely benefit is that you will learn something new about your diet and receive recommendations on possible ways to improve it.

**Will my taking part in this study be kept confidential?**

Information relating to your participation in the study will be kept confidential, and your rights to check personal data such as name, address, date of birth etc. will be fully protected in accordance with the Data Protection Act 1998. The results of the study will not be disclosed or published in any form by which you may be identified, unless your written permission is sought and obtained.

**Expenses and Remuneration**

Upon completion of the study you will be rewarded with £15 by the recruitment agency. Please make sure that you click the appropriate button at the end of each phase to take you back to Virtual Surveys.

**Who has reviewed the study?**

This study has been reviewed by an independent research ethics committee

**Thank you for considering whether you want to take part in this study**

By clicking the words I **AGREE** in the space below and by completing the attached survey I am giving my consent to participate in this study.

| | |
|---|---|
| 1. I confirm that I have read and understood the information for the above study. | Agree/Disagree |
| 2. I understand that my participation is voluntary and that I am free to withdraw at any time, without giving any reason, without my medical care or legal rights being affected. | Agree/Disagree |
| 3. I agree to take part in the above study. | Agree/Disagree |

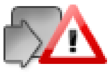

## APPENDIX 3 – QUESTIONNAIRES

Self-Report Index of Food (Margetts et al, 1989): **Stage 1/2**
**This questionnaire will be presented to subjects with the title '**Eating Habits'

Please choose the frequency with which you have eaten each of the foods in the list below.

| Food | 2 or more times per day | Every day | Three to five times per week | One to two times per week | One to three times per month | Rarely or never |
|---|---|---|---|---|---|---|
| Whole milk | | | | | | |
| Semi-skimmed milk | | | | | | |
| Skimmed milk | | | | | | |
| Butter | | | | | | |
| Margarine | | | | | | |
| Polyunsaturated margarine | | | | | | |
| Low fat spread | | | | | | |
| Ice cream | | | | | | |
| Yoghurt | | | | | | |
| Cheese- ordinary | | | | | | |
| Cheese- low fat | | | | | | |
| Eggs- fried | | | | | | |
| Eggs- not fried | | | | | | |
| Cheese/egg meals | | | | | | |
| Beef- roast/steak | | | | | | |
| Lamb- roast/chops | | | | | | |
| Pork- roast/chops | | | | | | |
| Chicken/Turkey | | | | | | |
| Bacon | | | | | | |
| Meat dishes | | | | | | |
| Canned meats | | | | | | |
| Meat pies/pasties | | | | | | |
| Sausages/beefburgers | | | | | | |
| Liver/Kidney/Pate | | | | | | |
| Fish- not fried | | | | | | |
| Fish-fried | | | | | | |
| Fish-canned | | | | | | |
| Bread-white | | | | | | |
| Bread-brown/granary | | | | | | |
| Bread-wholemeal | | | | | | |
| Sweet biscuits | | | | | | |
| Crackers/crispbread | | | | | | |
| Cakes/buns/pastries | | | | | | |
| Puddings | | | | | | |
| Breakfast Cereal-High fibre | | | | | | |
| Breakfast Cereal-Ordinary | | | | | | |
| Breakfast Cereal- Muesli | | | | | | |
| Rice/Pasta | | | | | | |
| Apples/Pears | | | | | | |

| | | | | | | |
|---|---|---|---|---|---|---|
| Oranges/Grapefruits | | | | | | |
| Bananas | | | | | | |
| Green Vegetables | | | | | | |
| Carrots/Tomatoes | | | | | | |

| **Food** | 2 or more times per day | Every day | Three to five times per week | One to two times per week | One to three times per month | Rarely or never |
|---|---|---|---|---|---|---|
| Other Vegetables | | | | | | |
| Baked beans | | | | | | |
| Other beans/lentils | | | | | | |
| Vegetable dishes | | | | | | |
| Potatoes- fried | | | | | | |
| Potatoes- not fried | | | | | | |
| Beer/lager | | | | | | |
| Wine/sherry/spirits | | | | | | |
| Tea/Coffee | | | | | | |
| Squash/Fizzy drinks | | | | | | |
| Low calorie drinks | | | | | | |
| Pure fruit drinks | | | | | | |
| Chocolate | | | | | | |
| Sweets | | | | | | |
| Sweet spreads | | | | | | |
| Sugar | | | | | | |
| Crisps and other savoury snacks | | | | | | |
| Nuts (including peanut butter) | | | | | | |
| Sauces and pickles | | | | | | |
| Salad oil/ dressing/mayonnaise | | | | | | |

Portion Changes: **Stage2**

**This question will be presented to subjects with the title** ‘Portion Changes’

During the last 4 weeks, my portions of the foods below were:

| A lot smaller | A lot bigger | | | | | | |
|---|---|---|---|---|---|---|---|
| | 1 | 2 | 3 | 4 | 5 | 6 | 7 |
| Meat dishes | | | | | | | |
| Cheese ordinary | | | | | | | |
| Cheese/egg meals | | | | | | | |
| Butter | | | | | | | |
| Whole Milk | | | | | | | |
| Bacon | | | | | | | |
| Beef- roast/steak | | | | | | | |
| Pork-roast/chops | | | | | | | |
| Lamb-roast/chops | | | | | | | |
| Chocolate | | | | | | | |
| Crisps and savoury snacks | | | | | | | |

Perceptions of saturated fat intake: **Stage 1/ 2**
**These two questions will be presented to subjects with the title '**Eating Habits'

| | Strongly Disagree | | | | | | Strongly Agree |
|---|---|---|---|---|---|---|---|
| I have eaten foods high in bad fats in the last four weeks | 1 | 2 | 3 | 4 | 5 | 6 | 7 |

| | Never | | | | | | Frequently |
|---|---|---|---|---|---|---|---|
| How often did you eat foods high in bad fats in the last four weeks? | 1 | 2 | 3 | 4 | 5 | 6 | 7 |

Maintenance Self-Efficacy: **Stage 1/2**
**This question will be presented to subjects with the title '**Keeping up the good work'

**How certain are you that you could overcome difficulties when trying to eat more healthily?**

| I am certain that I could overcome difficulties when trying to eat more healthily even if…. | Not at all 1 | Barely true 2 | Mostly true 3 | Exactly true 4 |
|---|---|---|---|---|
| I don't see success at once | | | | |
| I won't get support for my first attempts | | | | |
| It takes a long time to make it a habit | | | | |

Recovery Self-Efficacy: **Stage1/ 2**
**This question will be presented to subjects with the title** 'How do you feel about lapses?'

**In spite of good intentions, lapses or relapses may occur. If your eating have become unhealthy, how confident are you that you can return to a healthier diet?**

| I'm certain I could start eating a healthier diet again even after I had… | Not at all 1 | Barely true 2 | Mostly true 3 | Exactly true 4 |
|---|---|---|---|---|
| occasional lapses during the week | | | | |
| a full-blown relapse | | | | |

ACTION PLANNING: **Stage 2**
**These two questions will be presented to subjects with the title '**Planning'

| I now have my own plan regarding | Not at all | Barely true | Mostly true | Exactly true |
|---|---|---|---|---|
| When to eat a healthier diet | | | | |
| How to overcome my unhealthy eating habits | | | | |

COPING PLANNING: **Stage 1/2**

| Have you thought about possible barriers that could interfere with eating a healthier diet? | | | | |
|---|---|---|---|---|
| I have a detailed plan… | Not at all | Barely true | Mostly true | Exactly true |
| How to avoid high-risk situations where the urge to eat unhealthy food is high | | | | |
| How to arrange my daily routine to minimise the temptation to eat unhealthy food | | | | |

**FEEDBACK QUESTIONS Stage 2**
**These two questions will be presented to subjects with the title** 'Your Feedback'

| I found the information provided by the study so far: | | | | | | | |
|---|---|---|---|---|---|---|---|
| | Strongly Disagree | | | Strongly Agree | | | |
| Trustworthy | 1 | 2 | 3 | 4 | 5 | 6 | 7 |
| Of personal relevance to me | | | | | | | |
| Enjoyable | | | | | | | |
| Interesting | | | | | | | |
| Contained a lot of new information | | | | | | | |
| Credible | | | | | | | |
| Worrying | | | | | | | |
| A wake up call | | | | | | | |

Intentions: **Stage 1/2**
**This question will be presented to subjects with the title** 'Dietary Beliefs'

**Please rate your intentions to change the following aspects of your diet over the next two weeks.**

Over the next four weeks I intend to….

| | Strongly 1 | Strongly 2 | Disagree 3 | 4 | 5 | Agree 6 | 7 |
|---|---|---|---|---|---|---|---|
| Replace high fat with lower fat alternatives | | | | | | | |
| Eat smaller portions of high fat foods | | | | | | | |
| Reduce the amount of bad fats in my diet | | | | | | | |

General Health Behaviours: **Stage 2**
**This question will be presented to subjects with the title** 'Your General Health Behaviours'

**Compared to usual, over the last four weeks did you…..**

| | A lot less 1 | About the Same 2 | A lot more 3 | 4 | 5 | 6 | 7 |
|---|---|---|---|---|---|---|---|
| Drink alcohol | | | | | | | |
| Use Cholesterol lowering products | | | | | | | |
| Find you weigh | | | | | | | |

| | | | | | | | |
|---|---|---|---|---|---|---|---|
| Smoke | | | | | | | |
| Take up physical activity | | | | | | | |
| Eat a well-balanced diet | | | | | | | |
| Choose lower fat foods | | | | | | | |

## APPENDIX 4- Planning

**Slide 1**

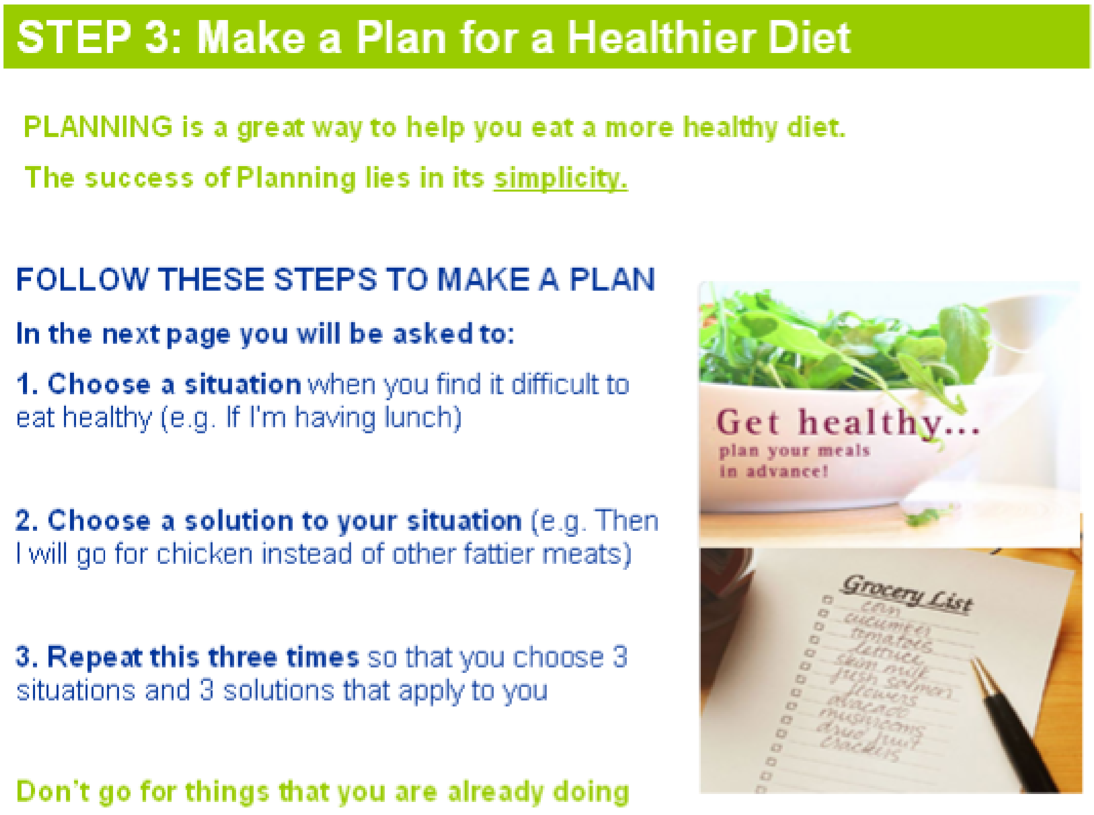


**Slide 2**

In this page you will set your **THREE PLANS**. Please follow instructions in the bubbles. It might take a few seconds for the system to load each selection. There are no right or wrong choices!

| Situations | | Solutions |
|---|---|---|
| If I go shopping | | Then I will choose a lower fat spread |
| If I go for a dish with cheese | | Then I will choose a low fat dairy product |
| If I'm getting a snack | | Then I will go for chicken |
| If I'm having lunch/dinner | | Then I will find out about a lower fat option |
| If I'm having breakfast | | Then I will go for oily fish |
| If I'm in a restaurant | | Then I will go for fruit instead of chocolate/crisps |
| If I'm having a good time with friends | | Then I will not buy high fat foods to keep in the house |
| If I'm feeling down or upset | | Then I will tell myself I can eat healthy! |
| If I'm craving a high fat food | | Then I will find something to distract myself |
| If I'm feeling hungry | | Then I will choose a lower fat cheese with it |
| If I had a hard day | | Then I will go for a smaller portion of the same food |

| | | |
|---|---|---|
| If others around me are eating high-fat foods | | Then I will go for a vegetable dish or soup |
| If I want to have a lamb or pork dish | | Then I will distract myself with something else |

**Slide 3**

# PLAN

This is a summary of your plan to follow over the next two weeks:

| Situations | Solutions |
|---|---|
| If I'm craving a high fat food | Then I will find out about a lower fat option |
| If I'm having breakfast | Then I will tell myself if I try hard I can eat healthily |
| If I had a hard day | Then I will go for fruit |

**REMEMBER:**

▪Choose **lower fat cheese** including low fat cheddar, edam light, cottage cheese, ricotta, philadephia.

▪Try to avoid sausages, roast, chops, meat pies and burgers. **Go for chicken or fish instead!**

▪**Distract** yourself with another activity.

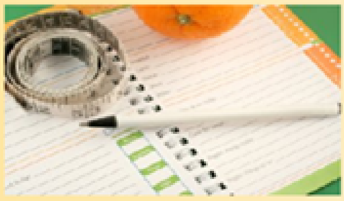

Please feel free to **PRINT THIS PAGE** for your records before clicking Next to continue the survey.

**NEXT**

## APPENDIX 5: Recruitment Agency Screening

# Health Check Survey: Draft Questionnaire v1
# 2417 – Email Invite

**Invite : Stage 1**

From xxxxxx xxxxxx

Subject: Help us with our research

Dear panel member,

Today, we are inviting you to take part in a Health Check survey focusing on healthy eating for a healthier cholesterol level.**This will involve interacting with the system at two times over a period of four weeks.** The first time will not take more than 30 minutes. All those eligible and completing all two different interactions will be paid <<pointsAsMoney>>

Instructions on how to take part can be found below. We look forward to hearing your opinions.

The full research programme will run until <<end_date>>. You can take part in the first stage until <<end_date>>. Please note this research project is limited to 725 respondents. Once 725 suitable respondents have completed the survey the project will be closed.

XXXX XXXX
**SSI**

**Invite : Stage 2**

Dear panel member,

Thank you for taking part in the Health Check survey which focused on healthy eating for a healthier cholesterol level. It is now time to take part in the second and final stage. All those completing both stages will be paid <<pointsAsMoney>>

Instructions on how to take part in the next stage can be found below. We look forward to hearing your opinions.

You can take part in the second and final stage until <<end_date>>.

XXXX XXXX
**SSI**

---

# Health Check Survey: Draft Questionnaire v1

# 2417

**Page 1: Welcome**
**Intro**
Thank you for your interest in this survey. **This will involve interacting with the system at two different times over a period of four weeks. Use of a printer and mobile phone might also be required.** Please can you answer the following profiling questions by clicking on a box for each. This profiling survey is being conducted by an independent market research agency in accordance with the Market Research Society Code of Conduct.

**Age**
[Ordered/Single]
Q1 What year were you born?
Answers

- Show as drop down list for the past 80 years (1927 to – last code – after 1991)
- List with most recent at the top

**SCREEN OUT IF BORN IN 1978 ONWARDS OR BEFORE 1949**
**Create a hidden variable which means that age is grouped into the following bands for quotas/monitoring:**

| | | |
|---|---|---|
| 29 or under | 1 | CLOSE |
| 30 to 45 years | 2 | |
| 46 to 60 years | 3 | |
| 61 or over | 4 | CLOSE |

**MaleFemale**
[Ordered/Single]
Q2 Are you male or female?
Answers

| | |
|---|---|
| Male | 1 |
| Female | 2 |

**Region**
[Ordered/Single]
Q3 Where do you live?
Answers

| | | |
|---|---|---|
| London/South East | 1 | |
| South West | 2 | |
| Eastern | 3 | |
| Midlands | 4 | |
| Wales | 5 | |
| Yorkshire & The Humber | 6 | |
| North West | 7 | |
| North East | 8 | |
| Scotland | 9 | |
| Northern Ireland | 10 | |
| Outside UK | 11 | CLOSE |

**Page 2: About You**

**PersonalInfo**
[Info]
**Note this information will be used for analysis purposes only and will not be attributed to you personally**

**Height**
[Ordered/Multi]
Q4 How tall are you?
Answers

| | | |
|---|---|---|
| Metres | 1 | OPEN |
| Don't Know | 99 | Excl |

**Weight**
[Ordered/Multi]
Q5 How much do you weigh?
Answers

| | | |
|---|---|---|
| Kilograms | 1 | OPEN |
| Don't Know | 99 | Excl. |

**Conversion Chart**
**Length**
1 inch = 2.5cm
1 foot = 30.48 cm
**Weight**
1 ounce = 28.35 grams
1 pound = 453.6 grams
1 stone = 6.3503 kgs

Q6 **BMI (HIDDEN QUESTION)**
[Ordered/Single]
Answers

| | | |
|---|---|---|
| <25 | 1 | Excl |
| 25 | 2 | |
| 26 | 3 | |
| 27 | 4 | |
| 28 | 5 | |
| 29 | 6 | |
| 30 | 7 | |
| 31 | 8 | |
| 32 | 9 | |
| 33 | 10 | |
| 34 | 11 | |
| 35 | 12 | |
| 36-40 | 13 | |
| 40+ | 14 | |

**Page 3 – About You**

**Smoker**
[Ordered/Single]

Q7 Which of these phrases best describes your smoking habits?
Answers

| | |
|---|---|
| Smoker | 1 |
| Non smoker | 2 |

**RANDOMLY ASSIGN SMOKERS TO EACH CELL**

**Heart**
[Random/Multi]
Q8 Which, if any, of the following have you ever been diagnosed with?
Answers

| | | |
|---|---|---|
| Diabetes | 1 | |
| Heart disease | 2 | CLOSE |
| High blood pressure | 3 | |
| Cancer | 4 | CLOSE |
| Any other chronic disease of the major organs (e.g. kidney failure etc.) | 5 | CLOSE / FIXED |
| Eating Disorder | 6 | CLOSE |
| None of these | 98 | |

**Cholesterol**
Q9 Are you motivated to eat a healthier diet and maintain a healthier cholesterol level? YES/NO (If no close)

Q10 Are you interested in joining a program that would help you eat a healthier diet and maintain a healthier cholesterol level? YES/NO (If no close)

Q11 What do you think your cholesterol level is?

a) Extremely low b) low c) somewhat low d) normal e) somewhat high f) high g) extremely high

Q12) Cholesterol score
a) My total cholesterol level …….mmol/L
I don't know
b) My HDL cholesterol level………mmol/L
I don't know

Q13) Has a healthcare professional e.g. GP, dietician ever told you that you have high cholesterol: YES/NO

Q14) Do you have a family history of high cholesterol? YES/NO

**Pregnant**
[Ordered/Single]
Q15 Are you pregnant at the moment?
Answers

| | | |
|---|---|---|
| Yes | 1 | CLOSE |
| No | 2 | |

**Mobile Use**
Q16 Do you have your own mobile phone? Yes/No (if no close)
Q17 Are you capable of opening text messages? Yes/No (If no close)

Q18 Are you willing to receive text messages? Yes/No (If no close)

## APPENDIX 6- Health Action Process Approach (HAPA)

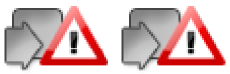

## <u>APPENDIX 7:</u> Participant Debrief

Dear Participant,

You have successfully completed this four week study with the aim to help you eat healthier for a healthier cholesterol level.

Would you like to be contacted to receive a FREE HEALTH PLAN to help you make positive changes to your diet and lifestyle?   **YES/NO**

YES     NO

Thank you for taking part in this study. For any further information please feel free to contact me on natasha.soureti@unilever.com

Best

Study-Co-ordinator

## <u>APPENDIX 8: EDUCATIONAL INFORMATION</u>

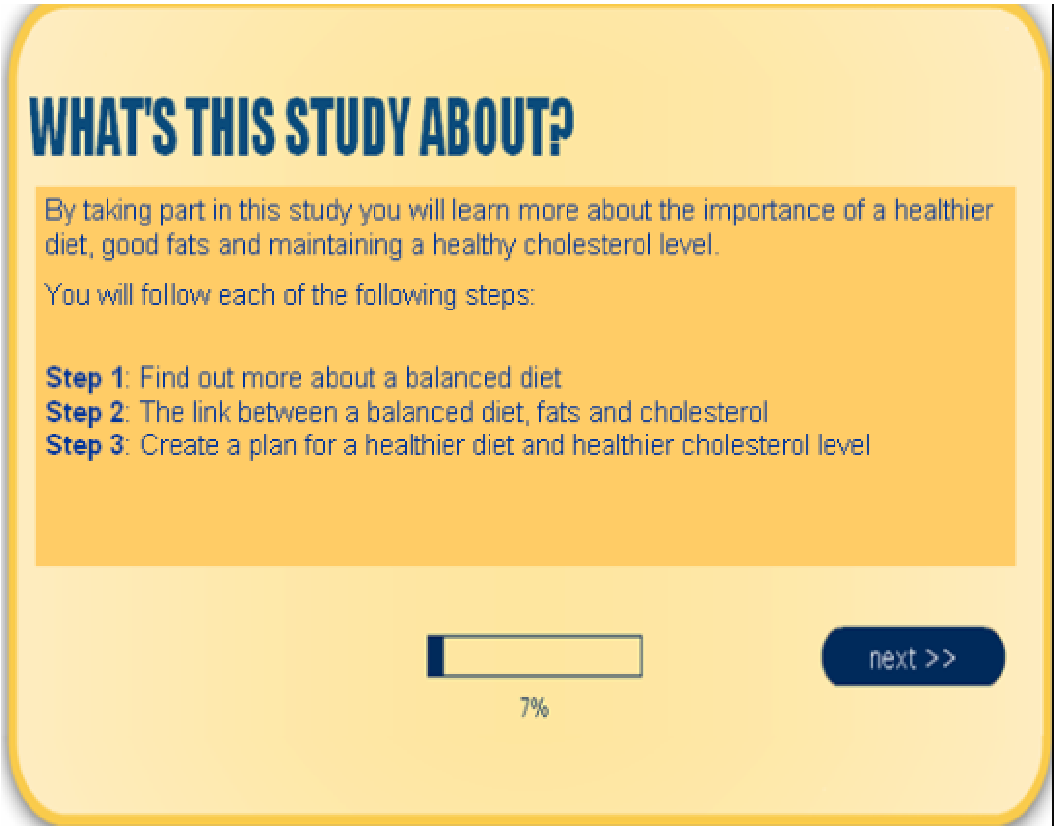
WHAT'S THIS STUDY ABOUT?
By taking part in this study you will learn more about the importance of a healthier diet, good fats and maintaining a healthy cholesterol level.
You will follow each of the following steps:
Step 1: Find out more about a balanced diet
Step 2: The link between a balanced diet, fats and cholesterol
Step 3: Create a plan for a healthier diet and healthier cholesterol level
next >>
7%

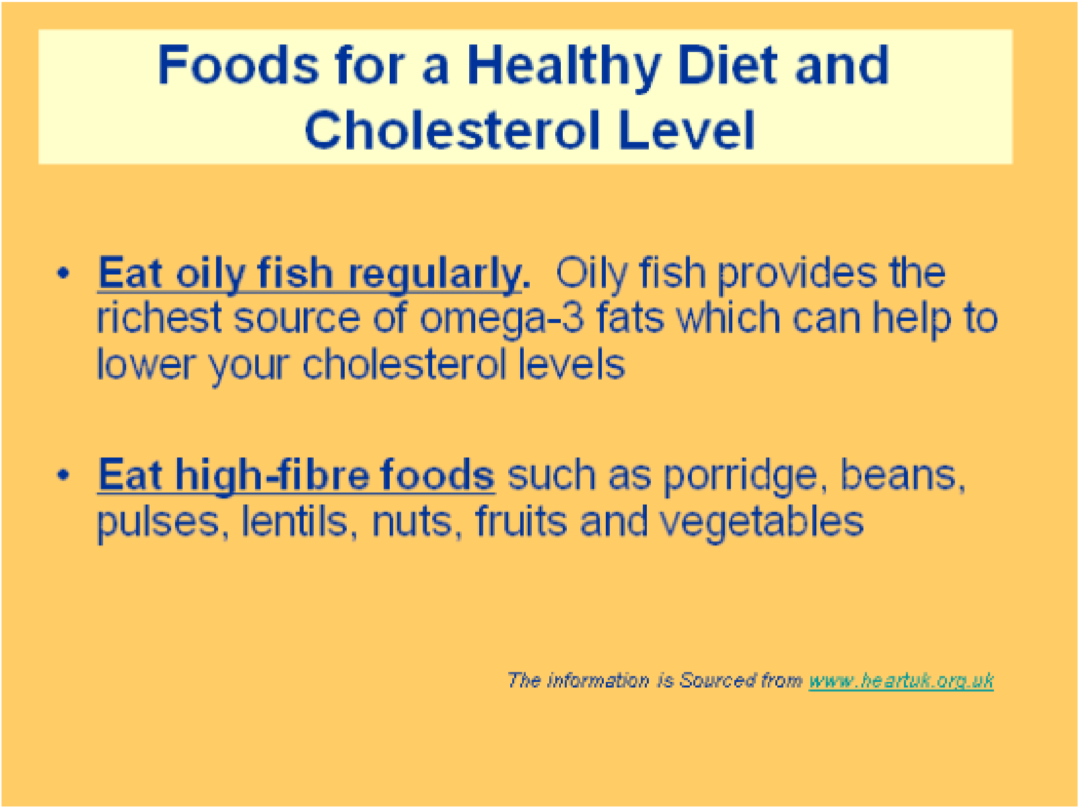
Foods for a Healthy Diet and Cholesterol Level
Eat oily fish regularly. Oily fish provides the richest source of omega-3 fats which can help to lower your cholesterol levels
Eat high-fibre foods such as porridge, beans, pulses, lentils, nuts, fruits and vegetables
The information is Sourced from www.heartuk.org.uk

# Bad Fats Increase Cholesterol

- What also matters in your diet is the type of fat you eat
- Eating too much of the wrong type of fat increases your cholesterol levels.
- For a healthy diet and healthy cholesterol level **cut down on the bad fats.**
- '**Bad**' fats = **saturated and trans fats-** include foods such as meat pies, sausages, butter, lard, hard cheese, cakes.

*The Information is sourced from the 'Eat Well, Be Well' Site by the Food Standards Agency and the 'Learn and Live' site by the American Heart Association.*

## IT'S NOT A MYTH- THESE ARE THE FACTS

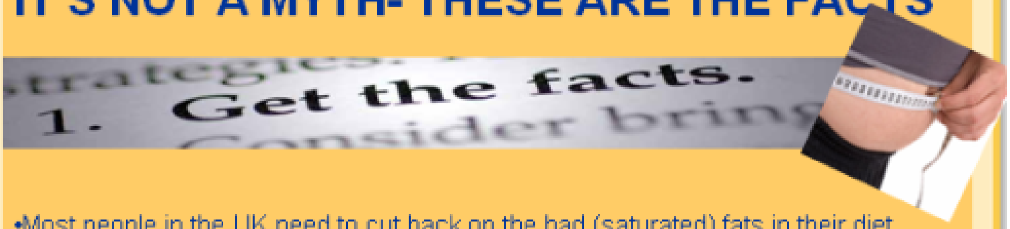


- Most people in the UK need to cut back on the bad (saturated) fats in their diet.
- If you are overweight then it is likely that you have a high cholesterol level

*The Information is sourced from the Eat Well, Be Well Site by the Food Standards Agency*

21%

next >>

# Plant Sterols help Lower Cholesterol Levels

- Plant sterols have been known for their cholesterol lowering properties for over 50 years
- When you go shopping look out for some **special products** where plant sterols are added such as low fat spreads (e.g.Flora ProActiv).

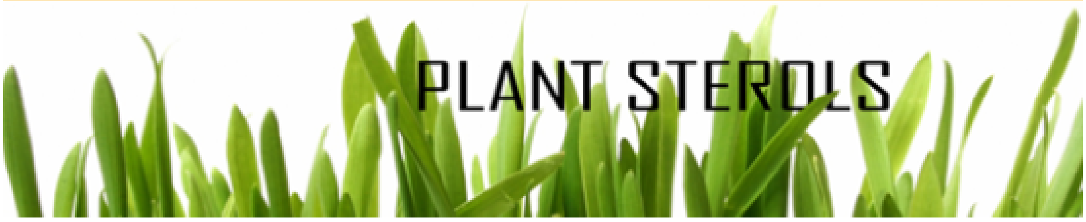


## TIPS FOR A HEALTHIER DIET

Below are some suggestions for a healthier diet, low in bad fats

**Eat chicken and oily fish more often**

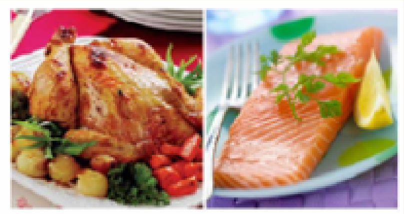

**Switch from butter to soft margarine**

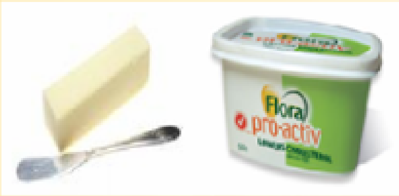


**Fill your plate with more salad & less meat**

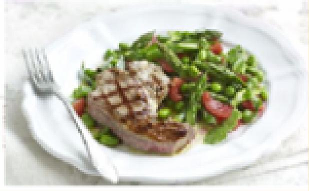

**Go for low fat dairy products**

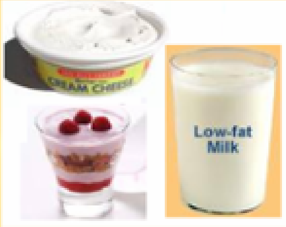

Supplement: Supplementary file 1 [file jmir_v13i4e118_app1.pdf]
