# Supplementary material for: Exploratory Study of Web-Based Planning and Mobile Text Reminders in an Overweight Population
Source: J Med Internet Res. 2011 Dec 20;13(4):e118. doi: 10.2196/jmir.1773 (PMC3278104; doi:10.2196/jmir.1773)
Supplement: Supplementary file 2 [file jmir_v13i4e118_app2.pdf]

Unilever Colworth
Sharnbrook
Bedford
MK44 1LQ
United Kingdom

T: +44 (0)1234
F: +44 (0)1234

From: Kathey Towler
On behalf of the CREC

To: Natasha Soureti

Date: 21st October 2009

Ethical Approval

Study Reference: Use of Planning and SMS Reminders in the Promotion of a Healthy Diet for a Healthier Cholesterol Level

Signed for the
Committee........K Towler........

Name........K Towler........

INVESTOR IN PEOPLE

Unilever UK Central Resources Limited
Registered number 29140
Registered office Unilever House,
Blackfriars, London EC4P 4BQ
